# Paddy: an evolutionary optimization algorithm for chemical systems and spaces†

**DOI:** 10.1039/d4dd00226a

**Published:** 2025-03-26

**Authors:** Armen G. Beck, Sanjay Iyer, Jonathan Fine, Gaurav Chopra

**Affiliations:** a Department of Chemistry, Purdue University 720 Clinic Drive West Lafayette IN 47907 USA gchopra@purdue.edu; b Purdue Institute for Drug Discovery West Lafayette IN 47907 USA; c Purdue Center for Cancer Research West Lafayette IN 47907 USA; d Purdue Institute for Inflammation, Immunology and Infectious Disease West Lafayette IN 47907 USA; e Purdue Institute for Integrative Neuroscience West Lafayette IN 47907 USA; f Regenstrief Center for Healthcare Engineering West Lafayette IN 47907 USA; g Department of Computer Science (by courtesy), Purdue University West Lafayette IN 47907 USA

## Abstract

Optimization of chemical systems and processes have been enhanced and enabled by the development of new algorithms and analytical approaches. While several methods systematically investigate how underlying variables correlate with a given outcome, there is often a substantial number of experiments needed to accurately model such relationships. As chemical systems increase in complexity, algorithms are needed to propose experiments that efficiently optimize the underlying objective, while effectively sampling parameter space to avoid convergence on local minima. We have developed the Paddy software package based on the Paddy field algorithm, a biologically inspired evolutionary optimization algorithm that propagates parameters without direct inference of the underlying objective function. We benchmarked Paddy against several optimization approaches: the Tree of Parzen Estimator through the Hyperopt software library, Bayesian optimization with a Gaussian process *via* Meta's Ax framework, and two population-based methods from EvoTorch—an evolutionary algorithm with Gaussian mutation, and a genetic algorithm using both a Gaussian mutation and single-point crossover—all representing diverse approaches to optimization. Paddy's performance is benchmarked for mathematical and chemical optimization tasks including global optimization of a two-dimensional bimodal distribution, interpolation of an irregular sinusoidal function, hyperparameter optimization of an artificial neural network tasked with classification of solvent for reaction components, targeted molecule generation by optimizing input vectors for a decoder network, and sampling discrete experimental space for optimal experimental planning. Paddy demonstrates robust versatility by maintaining strong performance across all optimization benchmarks, compared to other algorithms with varying performance. Additionally, Paddy avoids early convergence with its ability to bypass local optima in search of global solutions. We anticipate that the facile, versatile, robust and open-source nature of Paddy will serve as a toolkit in chemical problem-solving tasks towards automated experimentation with high priority for exploratory sampling and innate resistance to early convergence to identify optimal solutions.

## Abstract 2

Paddy is an algorithm implemented in Python for optimizing chemical systems. Benchmarked against Bayesian (Gaussian process, Tree-structured Parzen Estimator) and evolutionary optimization methods, Paddy shows excellent runtimes and robustness.

## Graphical abstract

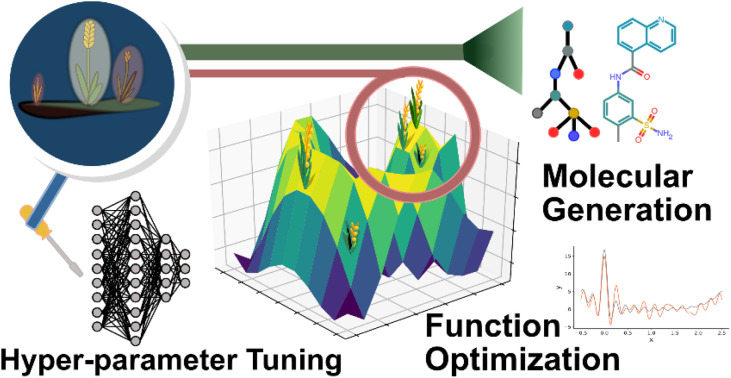

## Introduction

Optimization is used ubiquitously across the chemical sciences, from synthetic methodology,^1–3^ chromatography^4–6^ conditions, calculating transition state geometry,^7^ to selecting materials and drug formulations.^8–11^ Typically, several parameters or variables need to be optimized either by human chemists using chemical intuition or computational methods to identify suitable conditions.^12–14^ The development of automated optimization procedures for repetitive human tasks in chemical sciences, such as shimming,^15^ chromatograph peak assignment^16^ and developing bioanalytical workflows,^17^ have saved time and resources. Several chemical optimization methods have been used iteratively in a task-specific manner to optimize an objective to model or select experimental conditions for chemical and biological processes.^18–28^ However, iterations with stochastic optimization algorithms have been shown to provide an alternative to deterministic algorithms for finding optimal solutions.^29–32^ A well-known example is the use of stochastic gradient descent algorithms that outperform gradient descent.^33^ Several artificial intelligence and machine learning (AI/ML) architectures have been used in the chemical sciences where stochastic optimization algorithms are needed for train–validate–test cycles.^34,35^ These AI/ML algorithms are used in several areas of the chemical sciences, such as retrosynthesis,^36^ reaction condition prediction,^37–40^ catalyst design,^41,42^ drug design,^43–46^ spectral interpretation,^47–49^ retention time prediction,^50^ and for molecular simulations.^51–53^ Specific generative neural network architectures have also been used for inverse design^54,55^ and property-specific generation of molecules.^43,56–58^ For optimization tasks related to laboratory automation, that use closed-loop procedures, several methods have been developed^59^ including active learning using neural networks.^60,61^ In addition, the use of Bayesian methods,^62,63^ genetic algorithms^64^ and other iterative optimization methods^65^ have resulted in useful chemical outcomes without the use of prior learning.

Evolutionary algorithms are a class of optimization methods, inspired by biological evolution, that use a starting set of possible solutions (seeds) to the problem that are then evaluated using a ‘fitness (objective) function’ to iteratively ‘evolve’ a population of solution vectors towards optimal solutions. Using directed sampling to maximize a fitness function, evolutionary optimization algorithms propagate parameters to find the set of optimal solutions for a given problem. Several types of evolutionary algorithms include, genetic algorithms, evolution strategies, differential evolution, and estimation of distribution algorithms.^66^ The propagation between iterations use (meta)heuristic approaches with a set of rules that include simulated annealing,^67^ genetic algorithms,^68^ Tabu search,^69^ hill climbing methods,^70^ and particle swarm^66,71^ to name a few.

For evolutionary algorithms, it is primarily the development of selection and mutation operators,^72–74^ and genetic/crossover operators for genetic algorithms,^75^ that define their behavior and delineate them from each other. Additionally, to promote diversity or exploit successful solution space, methods called niching can leverage the density of solution vectors.^76^ In contrast to population-based evolutionary algorithms, Bayesian methods lend to directed optimization, guided by sequential updates of a probabilistic model and inferring the return on sampling, often *via* an acquisition function.^77^ Furthermore, Bayesian optimization methods have also been reported in the chemical literature for the optimization of neural networks,^39^ generative sampling,^78,79^ and as a general-purpose optimizer for chemistry.^62,80,81^ Generally, Bayesian optimization is favored when minimal evaluations are desired, as the computational costs may be considerable for larger and complex search spaces.

Herein, we have implemented a new class of evolutionary algorithm, the Paddy field algorithm (PFA)^82^ as a Python library, named Paddy. Paddy includes heuristic methods that operate on a reproductive principle dependent on solution fitness and the distribution of population density among a set of selected solutions. While evolutionary algorithms tend to differ based on how candidate solution vectors are selected and mutated or dispersed, it is the density-based reinforcement of solutions that distinguishes PFA. By considering the density of selected solution vectors, or plants, Paddy will have more offspring produced where higher densities of selected solution vectors are, in a step aptly named pollination. Unlike niching-based genetic algorithms, Paddy allows a single parent vector to produce a number of children, *via* Gaussian mutations, based on both its relative fitness and the pollination factor drawn from solution density. Additionally, a modified selection operator has been introduced with Paddy such that users can choose to only select and propagate from the current iteration and not the entire population, which can be beneficial for chemical optimization.

In this work we show the advantages of using Paddy, when compared to Bayesian-driven optimization^83^ implemented in the Hyperopt library^84^ and BoTorch^85^ using the Ax platform, and evolutionary and genetic algorithms using EvoTorch, with random solutions as controls. We compared test cases for accuracy, speed, sampling parameters and sampling performance across various optimization problems. Specifically, the problems include identification of the global maxima of a two-dimensional bimodal distribution, interpolation of an irregular sinusoidal function, hyperparameters optimization of a neural network trained on chemical reaction data, and comparison of performance for targeted molecule generation using a junction-tree variational autoencoder. Additionally, we demonstrate that Paddy can be used to optimally select experimental conditions. Overall, Paddy often outperforms or performs on par with Bayesian informed optimization and resulted in robust identification of solutions, with markedly lower runtime. Furthermore, Paddy was designed with user experience in mind, including features to save and recover Paddy trials. We provide complete documentation and code *via* GitHub (https://github.com/chopralab/paddy) to encourage others to use and extend Paddy for their chemical optimization tasks. We hope that chemists will find Paddy well suited for optimization across cheminformatic settings and mid to high-throughput experimentation.

## Methods

### Formulation of the Paddy field algorithm (PFA)

**Fig. 1:**
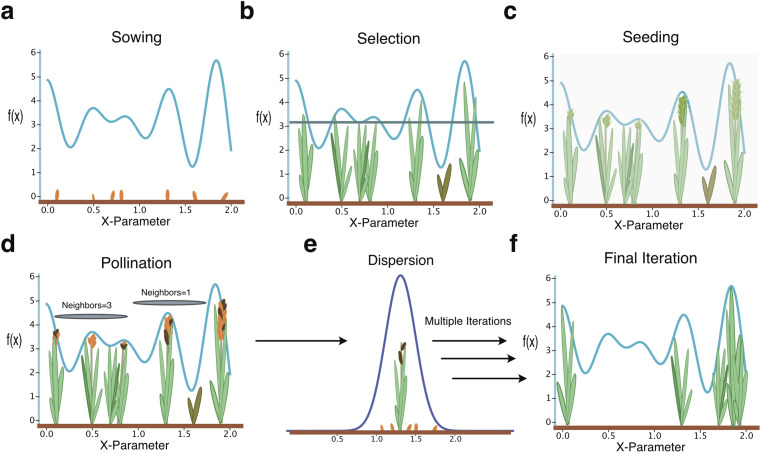
Overview of the Paddy field algorithm. Paddy is initiated by the sowing step (a) where objective function parameters, arbitrary in dimensionality, are randomly sown as the initial population of seeds. After evaluation of the seeds, the selection step (b) applies a selection operator to select a, user defined, number of top performing plants to further propagate. The seeding step (c) then calculates how many seeds a selected plant should respectively generate as to account for fitness across parameter space, such as fertility of soil determines the number of flowers a plant can grow. The pollination step (d) then reinforces the density of selected plants by eliminating seeds proportionally for those with fewer than the maximum number of neighboring plants within Euclidian space of the objective function variables. The sowing step (e) then assigns new parameter values to pollinated seeds by randomly dispersing across a Gaussian distribution, with the mean being the parameter values of the parent plant. The algorithm terminates (f) after converging or running for the number of iterations set.

The PFA was inspired by the reproductive behavior of plants that is based on the relationship of soil quality, pollination, and plant propagation to maximize plant fitness. The PFA proceeds without knowing this underlying relationship to iteratively optimize a fitness (objective) function using a five-phase process (a–e, Fig. 1). First, for any objective (fitness) function, *y* = *f*(*x*), with dependent parameters (*x*) of n-dimensions, PFA treats individual parameters *x* = {*x*_1_, *x*_2_, …, *x*_*n*_} as seeds to define a numerical propagation space. Next, these seeds are converted to plants by evaluating the objective (fitness) function, *y* = *f*(*x*), at the respective seed values. The resulting evaluation provides plant fitness score values, thereby assessing soil quality. Parameters (*x*_*H*_ ∈ *x*) that result in plants of high fitness (*y*_*H*_ ∈ *y*) are further evaluated and selected for seeding and propagation (y* ∈ *y*_*H*_). The number of neighboring plants and their fitness scores determine the number of seeds in each round (*s*) produced by a plant selected for propagation (y* ∈ *y*_*H*_), thereby directing plant density mediated pollination. The parameter values (x* ∈ *x*) for selected plants are then modified by sampling from a Gaussian distribution. We provide the details of the five-phase process (a–e) as follows:

(a) Sowing: the Paddy algorithm is initiated with a random set of user defined parameters (*x*), as starting seeds for evaluation. The exhaustiveness of this first step largely defines downstream processes involved in propagation of solution vectors. While very large sets of *x* will give Paddy a strong starting point, there is a cost tradeoff that should be considered. Conversely, lowering the number of seeds in *x* may hinder the exploratory behavior of Paddy. However, as the five-phase process repeats, the topology of the objective function and parameters will dictate the behavior of Paddy.

(b) Selection: the fitness function, *y* = *f*(*x*), is evaluated for the selected set of seed parameters (*x*), converting seeds to plants. A user-defined threshold parameter (*H*) that defines the selection operator which selects the number of plants based on the sorted list of evaluations (*y*_*H*_) for respective seeds (*x*_*H*_). These function evaluations can also be taken from previous iterations, for further propagation (eqn (1)).*f*(*x*) = *y* = {*y*_min_, …, *y*_max_},

1 *H*[*y*] = *H*[*f*(*x*)] = *f*(*x*_*H*_) = *y*_*H*_ = {*y*_t_, …, *y*_max_} ∀ *x*_*H*_ ∈ *x*, *y*_*H*_ ∈ *y*

where *y*_*H*_ is the sorted list of function evaluations (selected plants) from all current and previous evaluations *y* satisfying the threshold *H* for the set of seeds or parameters *x*_*H*_ that belong to all parameters *x*. Within subsequent sections of this manuscript, *y*_t_ denotes the integer value for the threshold parameter that defines the number of plants for selection.

(c) Seeding: the selected plants, y* ∈ *y*_*H*_, are used to calculate the number of potential seeds (*s*) for propagation as a fraction of user-defined maximum number of seeds (*s*_max_) given their min–max normalized fitness values (eqn (2)).

2 *s* = *s*_max_([y* − *y*_t_]/[*y*_max_ − *y*_t_]) ∀ y* ∈ *y*_*H*_

*s* is the number of seeds for selected plants (function evaluation) y* that belongs to the sorted (*y*_t_ minimum to *y*_max_ maximum) list of plants satisfying the threshold *y*_*H*_. Note that the Paddy software uses the variable *Q*_max_ in the code for what is denoted as *s*_max_ in this manuscript.

(d) Pollination: this step is related to clustering based on density of all selected plants y* ∈ *y*_*H*_ (function evaluation) such that the number of seeds to be dispersed by plants (new parameters *x* to be evaluated) is dependent on the number of neighbors to y*. The number of neighbors, *ν*, is used to calculate the pollination term *U* (eqn (3)) that ranges from 0.368 (inverse of Euler's number, *e*^−1^) to 1 (*e*^0^). The total number of pollinated seeds, *S*, to be subsequently propagated is the product of pollination term *U* and the number of seeds for selected plants *s* (eqn (4)).3
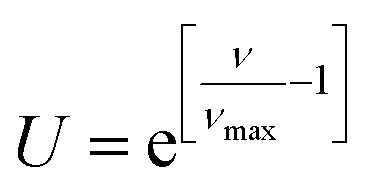

4 *S* = *U* × *s*

where the number of neighbors *ν* (eqn (5)) is defined as the number of selected plants or function evaluations y* = *f*(*x*_*k*_) ∈ *y*_*H*_ at *x*_*k*_ ∈ *x*_*H*_ within the radius (*r*) of the plant or function evaluation *f*(*x*_*j*_) being considered at *x*_*j*_ ∋ *x*_*k*_ ≠ *x*_*j*_ ∀ *x*_*i*_, *x*_*k*_ ∈ *x*_*H*_. When the absolute distance between plants (function evaluations) is less than the user defined hyperparameter *r*, they are all considered as neighbors (eqn (5)). To this end, the number of neighbors affects pollination term *U* in eqn (4) where term *U* = 1 for maximum number of neighbors *ν*_max_ and reduced to 0.368 (inverse of Euler's number, *e*^−1^) for no neighbors, *ν* = 0.

5 *ν* = |*n*|, *n* = {*x*_*k*_ ∈ *x*_*H*_|∥*x*_*j*_ − *x*_*k*_∥ − *r* < 0, y* = *f*(*x*_*k*_) ∈ *y*_*H*_, *x*_*k*_ ≠ *x*_*j*_}

(e) Dispersion: for each plant (function evaluation) with pollinated seeds, the parameter values for new seeds are initialized by sampling a Gaussian distribution where the parameter values of the parent plant define the mean of the Gaussian distribution for each parameter. The standard deviation (*σ*) is a hyperparameter that affects the dispersion of seeds (conditions) around each selected plant.

The steps a–e are then repeated until the desired number of iterations or specific termination conditions are met.

### Implementation and extension of the Paddy field algorithm

We have implemented the PFA algorithm and extended it with new features for chemical optimization problems. We have modified PFA^82^ such that the threshold parameter (*H*) may be adjusted based on the user defined random seeds during initiation. This allows for maximum flexibility in selecting seeds and threshold to allow for cases when the number of random seeds are lower than the threshold during initiation of PFA. Specifically, during initiation if the number of seeds is lower than threshold number to select the seeds for the next round, the value of *H* is equal to the rounded whole number of 75% of the number of random seeds defined by the user. In addition, the neighborhood function is modified in the pollination phase to mitigate early termination of the algorithm. For Paddy, we use Euclidean distance to determine the spatial distance between plants. The neighborhood function is dependent on the radius parameter that can result in early termination of the algorithm, in that the plants produce zero new seeds due to the user defined radius resulting in zero neighbors. To prevent unwanted terminations, we have formulated the neighborhood function with an adaptive radius to mitigate early termination. If the initial evaluation calculates zero neighbors, the 0.75th quantile for the distance between plants is used as the radius parameter. If the 0.75th quantile radius results in zero neighbors being assigned, the quantile value is iteratively decreased by 0.05 until a nonzero number of neighbors is assigned to a plant. If the 0.05th quantile fails to generate neighbors, each plant is evaluated as having one neighbor, effectively dropping the pollination term for the given iteration.

The termination condition is defined for equal values of *y*_t_ and *y*_max_. Additionally, in Paddy, the standard deviation parameter used for the dispersion phase is defined as 0.2. To provide flexibility to the user, modifications to the algorithm have been introduced in Paddy that facilitate alternative dispersion behavior, in addition to an alternative formulation of the selection phase which is described in the subsequent section.

We have introduced several alternative methodologies to provide users greater flexibility to control different features of the algorithm that include:

• Population mode: the selection phase is as described for the native PFA, where plants generated during any previous iteration are considered. As mentioned previously, population mode differs from the native PFA by having a flexible threshold parameter during, and only during, random initiation. The originally defined threshold parameter is recovered after the first iteration and remains static, as the full population of plants will remain available for propagation. If the selected threshold parameter is too large compared to the number of random seeds defined during initiation, population mode may not complete. This can result because the threshold parameter will not auto-scale for low numbers of plants post random initiation.

• Generational mode: the selection phase is modified such that only plants generated by the previous iteration are considered, rather than applying the threshold operator across all plants evaluated. A flexible threshold parameter is implemented as previously described, as some iterations may yield a number of seeds lower than the operator. The originally defined threshold operator is recovered and otherwise used each iteration.

• Scaled Gaussians: the standard deviation for the Gaussian applied during dispersion is calculated with an inherited scaling term (*δ*) (eqn (6)). The scaling term is initiated as zero and inherited in a variative manner where new values are generated by selecting from a Gaussian distribution, where the mean is the current scaling term and the standard deviation being 0.2.

6 *σ* = (0.2^10^)^*δ*^

• Parameter type: the parameter type determines the handling of values generated by Paddy where parameter types are either a continuous value or an integer value that is rounded after being generated.

• Parameter limits: the explicit bounding of a parameter value is supported by Paddy. Limits can be either one-sided or two-sided. If parameter values are generated outside set limits, they are clamped to the limit value.

• Parameter normalization: parameters with two-sided limits can be normalized during the dispersion phase *via* min–max normalization with limit values.

### Min/max optimization of a two-dimensional bimodal distribution

Determining the optimal min/max solutions is a fundamental problem in chemical sciences when the relationship between observable and dependent parameters are unknown. We evaluated the Paddy evolutionary algorithm, EvoTorch's evolutionary algorithm (EA) and genetic algorithm (GA),^86^ as well as two Bayesian optimization methods including BoTorch^85^*via* Meta's Ax framework and Hyperopt^84^ to find the maxima for a bimodal function using two parameters (*x*, *y*). Each algorithm was run 100 times with random initial seeds to test for robustness of the results. A solution was considered to have found the global maximum if it obtained a score greater than 0.81.

Hyperopt was run using the Tree-structured Parzen Estimator for 500 evaluations, and changed (*x*, *y*) parameters using ‘hp.uniform’ to propagate values between 0 and 1. Additionally, Meta's Ax framework was configured with a generation strategy that began with a Sobol sequence (SOBOL) for initial random exploration, conducting 200 trials with a maximum parallelism of 10, followed by Gaussian Process-based Expected Improvement (GPEI) for Bayesian optimization for the remaining 300 trials with a maximum parallelism of 20.

Paddy was run in generational mode with scaled Gaussian type setting and each (*x*, *y*) parameter limits of 0 and 1 that was randomly propagated with 0.01 resolution within the limits. ‘PFARunner’ parameters were set where: the number of random seeds as 50, the threshold number (*y*_t_) as 50, and the maximum number of seeds (*Q*_max_) as 100, radius (*r*) as 0.02, and iterations being 5.

The optimization was performed using EvoTorch with two different approaches: an evolutionary algorithm and a genetic algorithm. The searcher function was configured using the ‘GeneticAlgorithm’ class, which contained the ‘GaussianMutation’ operator for mutation and the ‘OnePointCrossover’ operator for crossover in the genetic algorithm setup. Both configurations used a population size of 200 and ran for 5 generations, with each experiment repeated 100 times. The Evolutionary Algorithm implementation used ‘GaussianMutation’ with a standard deviation of 0.2. The ‘GeneticAlgorithm’ instance additionally employed ‘OnePointCrossover’ with a tournament size of 4 for parent selection.

### Gramacy & Lee interpolation

Interpolation of parameters is an important problem in chemical sciences to robustly guide design of experiments conditions. We used this “toy example” as a representation to evaluate Paddy, Hyperopt, EvoTorch EA, EvoTorch GA, Ax, and the random search algorithm in the same manner as done for min/max optimization regarding environment, the number of executions, and random seeds. Interpolation of the Gramacy & Lee function was done using a 32^nd^ degree trigonometric polynomial with 65 coefficients values ranging between −1 and 1. Interpolative performance was evaluated by calculating the mean squared error between the Gramacy & Lee function and generated trigonometric polynomials, between −0.5 and 2.5 with a resolution of 0.001. The random sampling algorithm was used to generate the 65 coefficients using the NumPy ‘random.uniform’ function, with 5000 evaluations per execution.

Hyperopt was run using the Tree-structured Parzen Estimator for 1500 evaluations, and optimized the 65 coefficients using the ‘hp.uniform’ to propagate values between −1 and 1. Ax was run for 500 trials using Bayesian optimization with a Gaussian process model due to run time constraints.

Paddy was run in Generational mode with the Gaussian type set to default and with limits of −1 and 1, and randomly propagated in range of the limits with a resolution of 0.05. ‘PFARunner’ parameters were set where: the number of random seeds as 25, *y*_t_ as 25, *Q*_max_ as 25, *r* as 0.02, and iterations being 10.

The EvoTorch EA implementation utilized the ‘GeneticAlgorithm’ class with a population size of 250 and the ‘GaussianMutation’ operator set to a standard deviation of 0.2. The EvoTorch GA configuration also used the ‘GeneticAlgorithm’ class but included two operators: the ‘OnePointCrossOver’ operator with tournament selection size of 2 and the ‘GaussianMutation’ operator with standard deviation of 0.2, maintaining the same population size.

### Multilayer perceptron (MLP) hyperparameter optimization

Hyperparameter optimization is an important time-consuming problem to identify the best parameters for training neural networks. Training data was obtained from the Daniel Lowe^87^ repository (https://figshare.com/articles/dataset/Chemical_reactions_from_US_patents_1976-Sep2016_/5104873) and preprocessed. Briefly, the initial subset of reaction SMILES was generated by initially removing atom mapping, selecting reaction strings containing solely solvents as agents, and by associating ionic compounds with pseudo covalent bonds. Additionally, reactions with more than four reagents, post condensing of ionic pairs, or more than one product were removed. The resulting subset contains 4994 reactions with 30 types of solvents, and were converted into bitvectors after separating the reaction components from their respective solvent. The conversion to bitvectors was done using RDKit's ‘GetMorganFingerprintAsBitVec’ method^88^ to produce 2048 length Morgan Fingerprints^89^ using an atom radius of 2. The bitvectors and solvent labels were then converted into arrays, using one-hot encoding for solvent.

Machine learning was done using the PyTorch package for generating and training the neural networks, and the scikit-learn library for data splitting and performance assessment.^90,91^ A multilayer perceptron neural network architecture was built using Pytorch with two hidden layers. Each hidden layer included a linear layer followed by ReLU activation and a dropout layer. The input layer consisted of 2048 neurons, followed by the first and second hidden layers containing between 300 to 3000 and 32 to 2000 neurons, respectively. The output layer had 30 neurons. ‘CrossEntropyLoss’ was used as the loss function, which combines the softmax activation with the negative log-likelihood loss. The model was optimized using Adam, with a learning rate parameter (eps) set to 1 × 10^−7^.

The data preparation and loading process utilized PyTorch's ‘DataLoader’ to run the dataset in batches of 1000 during training and validation. For each fold in the stratified *k*-fold cross-validation, the training and validation sets were first wrapped in ‘TensorDataset’ objects, pairing the input features X with their corresponding labels Y. These datasets were then passed to ‘DataLoader’ instances with a batch size of 1000. The training data loader used shuffling to randomize the order of samples in each epoch.

Stratified *k*-fold validation was used of three-fold splitting of training and validation data. Models were trained for five epochs, using batch sizes of 1000, with validation scores being calculated as micro F1 scores. The F1 scores of the three resulting models post three-fold cross validation were averaged to provide a single value for the algorithms to optimize.

The hyperparameters of the two dropout terms, ranging from 0–1, and the lengths of the hidden layers, 300–3000 and 32–2000 neurons for the first and second layers respectively, were each optimized over 100 trials using Paddy, Hyperopt, Ax, EvoTorch EA, EvoTorch GA, and a random search algorithm. The random search algorithm generated random dropout terms and layer lengths using the NumPy ‘random.uniform’ and ‘random.randint’ functions, to propagate values for the dropout and layer length terms within their appropriate ranges, for 200 evaluations.

Hyperopt was run with the Tree-structured Parzen Estimator for 150 evaluations, and used the ‘hp.uniform’ and ‘hp.quniform’ functions for dropout and layer length value generation. Ax was configured to perform Bayesian optimization of the MLP's hyperparameters using the optimize function to maximize the F1 score over 150 trials.

Paddy was run in Generational mode with the Gaussian type set to default and using normalization when generating parameter values. Random propagation was done with dropout values between 0–0.5 with 0.05 in resolution and layer lengths of 300–3000 and 32–500 with resolutions of 0.05 for the first and second hidden layers, and propagated within the parameter limits for subsequent iterations. ‘PFARunner’ parameters were set where: the random seed number as 25, *y*_t_ as 5, *Q*_max_ as 10, *r* as 0.2, and iterations being 7.

EvoTorch was configured with an initial hyperparameter search space bounded by lower limits of [500, 0.0, 32, 0.0] and upper limits of [1000, 0.5, 500, 0.5]. This search space was subsequently expanded to lower bounds of [300, 0.0, 30, 0.0] and upper bounds of [3000, 1, 2000, 1]. For EvoTorch's EA, ‘GeneticAlgorithm’ was initialized with a population size of 20 and employed the ‘GaussianMutation’ operator with a standard deviation of 0.2 to explore the hyperparameter space. Additionally, EvoTorch's GA utilized the same ‘GeneticAlgorithm’ class and population size but also incorporated a ‘OnePointCrossOver’ operator with a tournament selection size of 3 alongside the ‘GaussianMutation’ operator.

### Junction tree variational autoencoder (JT-VAE) latent space sampling

The JT-VAE pretrained models are available on GitHub (https://github.com/wengong-jin/icml18-jtnn) that includes the Conda environment and all Python 2 dependencies. Since JT-VAE requires Python 2 environment, both Ax and EvoTorch were not benchmarked. Using fixed random seeds to ensure reproducibility, latent vectors were decoded to generate SMILES strings prior to evaluation. Fitness calculated using solely Tversky Similarity, was done using the RDKit library, by converting SMILES to RDKit mol structures and subsequently to Morgan Fingerprints. The Morgan Fingerprints were generated with a bit radius of two, and length of 2^23^ to minimize bit collision incidents, *via* the ‘GetMorganFingerprintAsBitVect’ method. The Morgan Fingerprints of the generated SMILES and Pazopanib molecule were then compared *via* Tversky Similarity with coefficients *α* = 0.5 and *β* = 0.01. The *α* and *β* coefficients were used to scale the relative complements of the Pazopanib fingerprint in the generated fingerprint and vise versa respectively. For the random sampling algorithm, it generated the tree and graph latent vectors as two arrays, with a length 28 and with values between −1 and 1, for 3500 evaluations. Hyperopt was run similarly, generating the 56 values between −1 and 1 using the ‘hp.uniform’ function, and set to evaluate 3500 times using the Tree-structured Parzen Estimator. Paddy was run in both Generational and Population mode, with the Gaussian type set to scaled and with limits of −1 and 1, and randomly propagated in range of the limits with 0.05 in resolution. ‘PFARunner’ parameters were set with the random seed number as 250, *y*_t_ as 15, *Q*_max_ as 25, *r* as 5, and iterations being 30.

The trials were run using the multi-feature custom metric by using the same parameters for Tversky Similarity. A custom metric was developed using methods in the RDKit library by modifying terms of the target chemical property function described in the JT-VAE manuscript.^92^ Briefly, the target chemical property function, defined by Jaakkola *et al.*, is the difference between the octanol–water partition coefficient (log *P*) of a molecule, with the Synthetic Accessibility (SA) score and number of cycles with an atom count greater than six (defined as cycle variable) (eqn (7)). For our custom metric, we incorporated SA and cycle in addition to Tversky Similarity (TV), fingerprint density (FD), number of rotatable bonds to define Rotatable Bond Score (RBS), the number of cycles to define Cycle Count Score (CCS), and number of on bits to define Bit On Score (BOS) (eqn (8)).

Tversky Similarity was calculated using the same parameters as described previously. Fingerprint density was calculated using the RDKit ‘FpDensityMorgan3’ function, which generates Morgan Fingerprints as undefined integer sparse bit vectors with a bit radius of three and returns the quotient of on bits by the number of non-hydrogen atoms in the molecule. Fingerprint density was used to promote structurally diverse molecules. Rotatable bonds were enumerated for molecules *via* RDKit, and used in a conditional manner to calculate a Rotatable Bond Score (RBS, see eqn (9)), and penalize long chain and flat molecules. The cycle calculation was expanded as to provide a conditional Cycle Count Score (CCS, see eqn (10)) to promote the generation of molecules between two and five rings. The RDKit method ‘GetMorganFingerprintAsBitVect’, which generates explicit bit vectors was employed with previously described parameters, with the number of on bits (mb) used to penalize molecules with less than 45 on positions as the Bit On Score (BOS, see eqn (11)).

7 *f*(*m*) = log *P* − SA − cycle

8
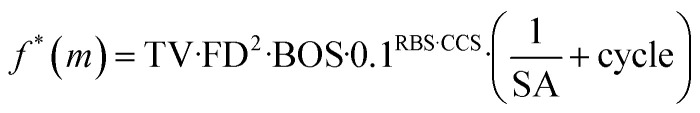
9
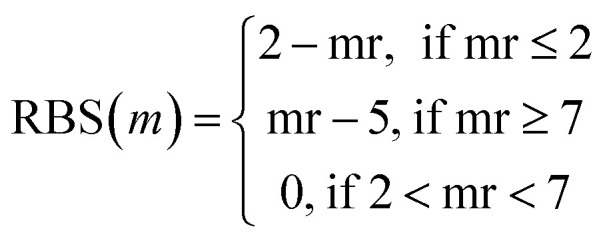
10
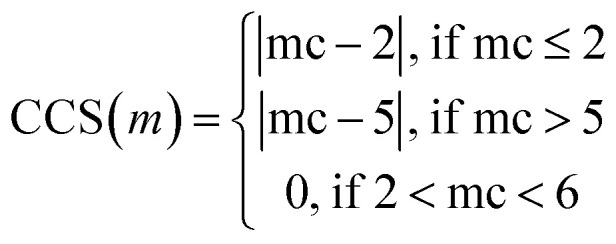
11
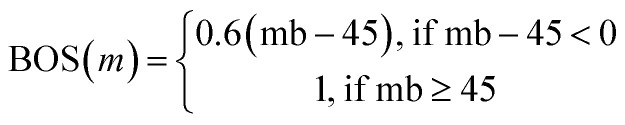
mr, rotatable bonds in a molecule *m*. mc, number of cycles in a molecule *m*. mb, number of on bits in an explicit bit vector for a molecule *m*.

### Visualization of latent space

Libraries used for analysis and visualization, were UMAP and Matplotlib respectively.^93^ The 56 values in the latent vectors generated by Paddy were used as input features. The 56 length vectors were then reduced *via* UMAP to three components, while setting the number of neighbors to 15 and minimum distance between projected points to 0.5. All other parameters were set to default values.

### Simulated optimization of experimental conditions

Using experimental data from ligand-based pharmacokinetic screening published in partnership with the pharmaceutical company Merck Sharp & Dohme (MSD),^17^ we utilized Paddy for the simulated optimization of assay conditions. This was done where Paddy would optimize four experimental conditions: biotinylated anti-idiotypic (anti-ID) antibody, Sulfo-Taged anti-ID, the assay format, and concentration of antigen. With these conditions being categorical in nature, they were managed using the ‘NumericAssistant’ from the Paddy utils library. This enables Paddy to sample from a continuous parameter space while returning the index of nearest discrete conditions. Additionally, the ‘NumericAssistant’ allows for unique experiments, in that the same condition cannot be selected more than once. After each round of experiments, the metric values of experiments selected by Paddy are added to a pool for updating the objective function between iterations. As the number of iterations increases for Paddy, experiment index values are used to recalculate and normalize the metric values for the selected experiments. Normalized scores are updated within the ‘PaddyRunner’ class prior to executing the PFA, ensuring no information leakage from unseen experimental values. A grid search across multiple *y*_t_ and *Q*_max_ values was performed to evaluate their impact on performance.

### Paddy parameter grid searches

Grid searches were performed to evaluate the *Q*_max_ and *y*_t_ parameters from the Paddy algorithm on the Gramacy–Lee and MLP benchmarks. Gramacy–Lee grid search used *Q*_max_ values 20, 25, 30, 40, 50, 75, 100, 200, 500, 1000 and *y*_t_ values 10, 15, 20, 25, 30. The MLP grid search used *Q*_max_ values 10, 15, 20, 25, 30, 35, 40 and *y*_t_ values 3, 4, 5, 6, 7. Each combination was evaluated with five repeats for both benchmarks. All other parameters matched those used by the Paddy algorithm in the Gramacy–Lee and MLP benchmarks methods section.

### Hardware

All Gramacy Lee and MLP benchmarks were conducted on Purdue University's high-performance computing cluster (HPC) Gilbreth, specifically run on “Node K”. Each Node K features 52 CPU cores with 512 GB system memory, 2 Nvidia A100 GPUs with 80 GB of GPU memory per card. The node is interconnected *via* 100 Gbps Infiniband, running on CentOS 7 with Slurm batch scheduling.

The JT-VAE experiments were conducted on FP001, a Chopra Lab server, which is equipped with an Intel Xeon E5-2690v4 processor running at 2.60 GHz with 56 cores and two Nvidia GTX 1080 GPUs.

## Results and discussion

### Paddy identifies correct global maxima of a two-dimensional bimodal distribution

**Fig. 2:**
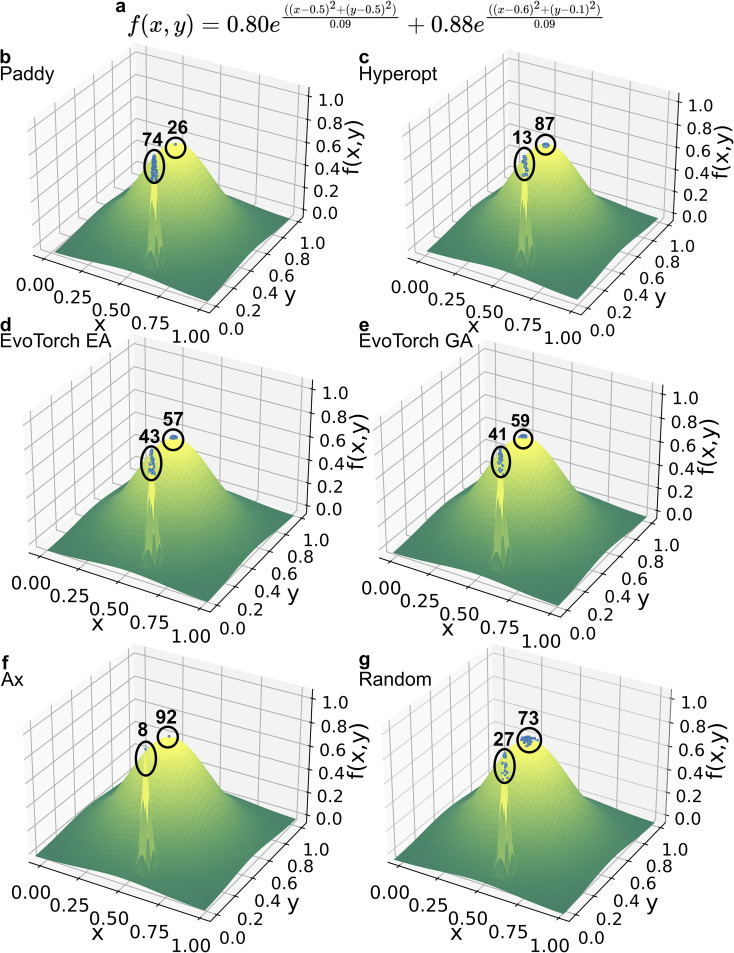
Global optimization of unknown function example as (a) 2D bimodal distribution *f*(*x*, *y*) with a local maxima at (0.5, 0.5) and a global maxima at (0.6, 0.1). Comparison of global maxima identification success rates across optimization algorithms over 100 runs without knowledge of the underlying mathematical function: (b) Paddy (74%), (c) Hyperopt (13%), (d) EvoTorch EA (43%), (e) EvoTorch GA (41%), (f) Ax (8%), and (g) random search (27%).

We employed a two-dimensional bimodal distribution function with two parameters (*x*, *y*) to assess the performance of Paddy to identify the global maximum, out of two maxima (Fig. 2a). The slope of the global maximum is steeper than that of the local maximum, presenting a challenge for global optimization as there is a greater probability that initial sampling will occur near the local maximum with rare events at the global maxima. Paddy, Hyperopt, Ax, EvoTorch EA, EvoTorch GA, and random were evaluated 100 times, with different starting conditions. The global maxima was found in 74 trials with Paddy, 43 with EvoTorch EA, 41 with EvoTorch GA, 13 with Hyperopt, 8 with Ax and 27 with random (Fig. 2b–g). These results suggest that evolutionary and genetic algorithms may be a more effective approach than Bayesian-based algorithms for identifying rare experimental events.

### Interpolation of the Gramacy & Lee function using Paddy

To showcase the use of optimization problems to efficiently sample several parameters, we used interpolation of the Gramacy & Lee function^94^ using a 65^th^ degree trigonometric polynomial, as an example to showcase a possible future application in parameter selection for design of experiments. The performance was evaluated as the mean squared error (MSE) between the *y* values generated by the 65 fitted polynomial coefficients and the Gramacy & Lee objective function, where *x* ∈ [−0.5, 2.5] and with a resolution of 0.001.

**Fig. 3:**
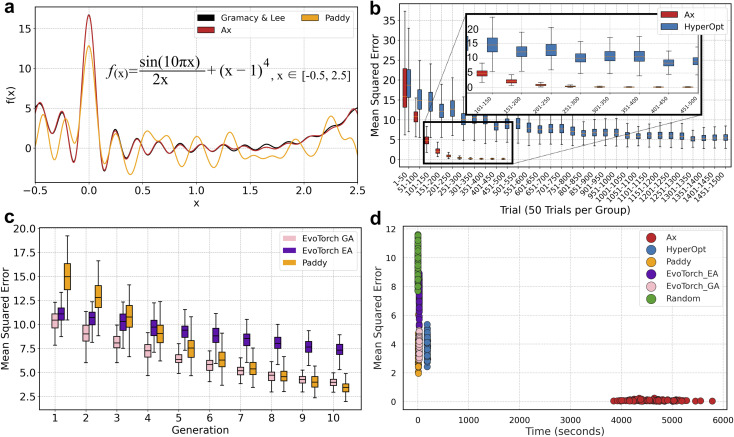
Interpolation of (a) Gramacy & Lee function overlaid with the best results from each algorithm. (b) Box plot showing the distribution of mean squared error (MSE) scores for the Bayesian optimization algorithms Ax and Hyperopt across 500 and 1500 trials respectively. Trials are grouped in bins of 50 on the *x*-axis. (c) Box plot of MSE values across population-based algorithms over 10 generations, with 100 repeats on the Gramacy & Lee benchmark. Compared are Paddy, EvoTorch using an evolutionary algorithm (EA), and EvoTorch using a genetic algorithm (GA), displayed in orange, purple, and pink respectively. (d) Scatter plot of best MSE scores (*y*-axis) *vs.* run time (*x*-axis) for 100 repeats across six algorithms.

To assess the robustness of the algorithms, we evaluated the performance of Paddy, Hyperopt, Ax, EvoTorch EA, EvoTorch GA, and random sampling optimizations for 100 different runs. The population-based algorithms evaluated the function over 10 generations, while the Bayesian methods Hyperopt and Ax explored the search space over 1500 trials and 500 trials, respectively. Random sampling was conducted with 1500 trials for a baseline assessment. Fig. 3 illustrates the best MSE value achieved in each generation by the population-based algorithms. Although Paddy started with a higher initial MSE compared to EvoTorch GA and EvoTorch EA, it rapidly improved to match EvoTorch GA's performance by generation four and surpassed EvoTorch by generation five. Although EvoTorch GA remained competitive, it never exceeded Paddy's performance in subsequent generations.

Paddy achieved lower MSEs compared to Hyperopt, EvoTorch EA, EvoTorch GA, and the random sampling algorithm. However, Ax produced the best fit interpolations, with an average MSE of 0.085. These results were validated visually by plotting interpolated functions (Fig. 3a and S1–6†). While Ax achieved greater performance on this benchmark, it required an average of 4595.75 s per repeat. In contrast, Paddy required 10.33 s per repeat, an approximately 444 times faster runtime. Other population-based algorithms, EvoTorch GA and EvoTorch EA, also exhibited rapid runtimes with average runtimes of 25.25 and 25.55 s, respectively. Meanwhile, Hyperopt maintained an average runtime of 185.06 s reflecting the slower performance observed among Bayesian optimization methods for the Gramacy Lee benchmark. Lastly, relationship between the *y*_t_ and *Q*_max_ parameters and Paddy performance was investigated, with higher values found to trend with decreased MSE, with an *y*_t_ and *Q*_max_ of 25 and 500 resulting in an MSE of 0.60 (Fig. S7†).

These results highlight Paddy's light-weight computation and performance, making it a suitable choice when low-cost experimentation can be exploited (Table 1).

**Table 1:** Performance of on Gramacy & Lee Benchmark^a^

Algorithm	Minimum MSE	Mean MSE	Mean repeat time (s)
Ax	0.019	0.086 ± 0.043	4595.75 ± 400.69
Paddy	1.962	3.441 ± 0.651	10.33 ± 0.48
EvoTorch (EA)	5.240	7.311 ± 0.812	25.55 ± 0.13
EvoTorch (GA)	2.934	3.939 ± 0.443	25.25 ± 0.50
Hyperopt	2.421	3.908 ± 0.602	185.06 ± 1.96
Random	7.688	9.661 ± 1.010	0.80 ± 1.48

a ± root mean squared error.

### Paddy supports hyperparameter optimization of a multilayer perceptron

**Fig. 4:**
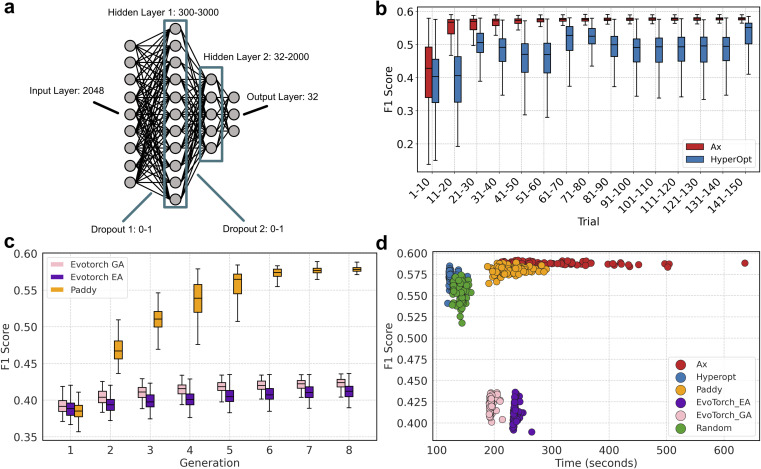
Hyperparameter optimization. (a) Optimization of hyperparameters for a neural network trained to predict solvent for the Morgan Fingerprint of reaction components. The architecture of the neural network contained two hidden layers, with the length and dropout of each layer being the objective function variables to optimize. (b) Box plot showing the distribution of F1 scores for the Bayesian optimization algorithms Ax and Hyperopt across 150 trials. Trials are grouped in bins of 10 on the *x*-axis. (c) Box plot of F1 scores values across population-based algorithms over 8 generations, with 100 repeats for the optimization of a multilayer perceptron. Compared are Paddy, EvoTorch using an evolutionary algorithm (EA), and EvoTorch using a genetic algorithm (GA), displayed in orange, purple, and pink respectively. (d) Scatter plot of best F1 scores (*y*-axis) *vs.* run time (*x*-axis) for 100 repeats across six algorithms.

The AI/ML architectures, such as artificial neural networks have been used extensively in cheminformatics, bioinformatics, and computational chemistry/biology in recent years.^34,35,46,95^ However, training large AI/ML models present a major challenge for lowering computational costs for training/validation to efficiently select hyperparameters^96^ while maintaining performance of the models. To showcase an example of Paddy for efficient use of hyperparameter optimization, we used a multiplayer perceptron (MLP) with two hidden layers (Fig. 4a). This MLP was designed as a multiclass classifier trained to classify reactions by selecting suitable solvent, such that the reaction inputs were represented as Morgan Fingerprints were trained with the output for one of 32 solvent labels. The average F1 score resulting from 3-fold cross validation was used as the objective function to sample hyperparameters. Specifically, we assessed the performance of Paddy, Hyperopt, Ax, EvoTorch EA, EvoTorch GA, and random search to select the number of neurons and the dropout rate for the two hidden layers. The number of neurons is an integer in the range of 300–3000 neurons for the first layer and 32–2000 neurons for the second layer. Dropout is a real number between 0 and 1. The ability for Paddy to confine the values used during random initiation was employed to apply these constrains, with dropout values from 0–0.5 and lengths of 500–1000 and 32–500 neurons for the first and second hidden layers respectively. To showcase robustness of the method, 100 trials of each method were done, and we found both Paddy, Ax, Hyperopt outperformed the random sampling algorithm, while EvoTorch EA and GA did not (Table 2).

**Table 2:** Performance on multilayer perceptron hyperparameter optimization benchmark^a^

Algorithm	Maximum F1 score	Minimum F1 score	Mean F1 score	Mean repeat time (s)
Ax	0.592	0.584	0.588 ± 0.002	296.89 ± 75.30
Paddy	0.589	0.565	0.580 ± 0.004	232.22 ± 22.47
EvoTorch EA	0.436	0.390	0.413 ± 0.011	236.12 ± 4.21
EvoTorch GA	0.436	0.401	0.423 ± 0.008	195.01 ± 3.85
Hyperopt	0.585	0.541	0.574 ± 0.006	124.08 ± 4.48
Random	0.574	0.518	0.555 ± 0.011	140.36 ± 6.64

a ± root mean squared error.

In this benchmark (Fig. 4b–d), both Bayesian-based methods demonstrated strong performance, with Ax achieving the highest average F1 score of 0.588 and Hyperopt with 0.574, confirming the effectiveness of Bayesian approaches for MLP hyperparameter optimization. Notably, Paddy achieved an average F1 score of 0.580, which was 0.008 less than Ax, whereas other evolutionary approaches, EvoTorch's evolutionary and genetic algorithms, both attained markedly lower scores.

In the population-based algorithms, while the initial generation exhibited comparable performance scores across all three algorithms, Paddy demonstrated a significant performance increase by generation 2 (Fig. 4c), quickly surpassing the EvoTorch algorithms. In subsequent generations, the performance disparity between Paddy and the other population based algorithms became increasingly pronounced with Paddy achieving a mean F1 score of 0.580 (±0.004), outperforming EvoTorch EA and EvoTorch GA by 40.4% and 37.1% respectively (0.413 ± 0.011 and 0.423 ± 0.008). Again, when concerning runtime, Paddy outperformed Ax, with a 22% lower runtime on average. When testing the performance of Paddy in relation to *y*_t_ and *Q*_max_, the choice of parameterization again favored greater values, and was able to yield F1 scores of 0.590 under multiple conditions (Fig. S8†).

Overall, these results highlight Paddy's versatility. While it shares evolutionary principles with EvoTorch's algorithms, it performs comparably to Bayesian optimization methods for hyperparameter tunning. Additionally, while Paddy produced F1 scores marginally lower than Ax, runtime was significantly lower, again supporting its suitability for low-cost experimentation.

### Sampling latent space with Paddy for targeted molecule generation

Another popular application of AI/ML models in chemical sciences is the use of generative neural networks,^97^ where the model learns the mapping between input and output from random inputs. These models are then used to generate desired outputs satisfying specific conditions such as experimental conditions^98^ or molecular structures^43,54,58^ based on the mapping of random input to the desired output from the training set. For the task of molecule generation, a popular neural network architecture employed are encoders and decoders.^57,58,99,100^ An example of encoder/decoder architecture is an autoencoder, a neural network that is trained to reduce dimensionality of an input and subsequently generate an output, that is ideally, identical to the initial input.^101^ The portion of the network tasked with dimension reduction is the encoder network, and the network that reconstructs the input being the decoder network. Transient values feed forward between the encoder and decoder, are often referred to as either latent representations or latent variables. Once trained, autoencoders can then be used in a generative manner by providing a latent representation as input to the decoder network. Furthermore, targeted generation can be conducted *via* sequential optimization of a latent vector, with examples of this in drug discovery.^43,57^

While latent representations have been used for non-generative tasks, the variational autoencoder (VAE) has emerged as an architecture particularly well suited for generative tasks. This is due to the latent variables of VAEs being regularized, as VAEs are trained to optimize the parameters for encoding normal distributions and subsequently decode from latent vectors propagated from these distributions. Regularization of the latent space is further reinforced such that the learned distributions are trained to fit a standard normal distribution, with a mean of 0 and standard deviation of 1, in conjunction to input reconstruction fidelity. The regularization of latent distributions results in continuous latent spaces with minimized sparsity. Due to these features, VAEs are well suited for generative tasks, where latent space sampling often generates outputs similar in nature to those of neighboring latent features.^102^

**Fig. 5:**
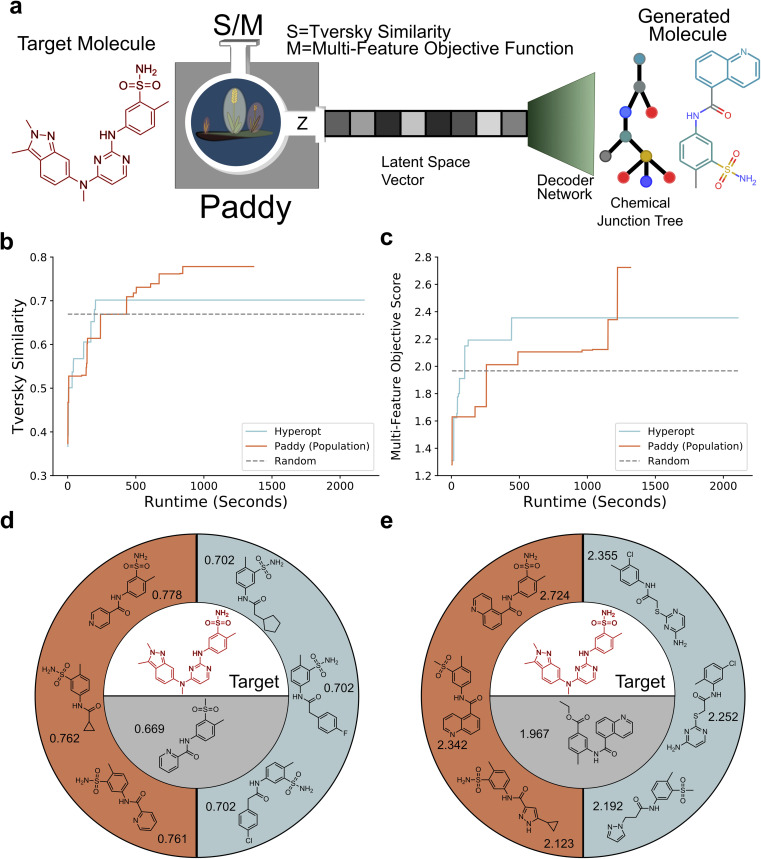
Overview of Paddy JT-VAE pipeline (a), where latent space vectors are optimized, such that when decoded to chemical junction trees, generating molecules to maximize an objective function that incorporates a target molecule (Pazopanib). Tversky Similarity (b) and a multifeatured objective function (c) trials are plotted as the running solution over runtime for Paddy and Hyperopt, where the highest scoring random search solution is plotted as a dashed line for comparison. The top three molecules generated by Paddy (orange) and Hyperopt (blue) for the Tversky Similarity (d) and multifeatured objective function (e) trials are displayed with their respective scores, with Pazopanib in red and the random search solution bellow (gray).

To showcase the ability of Paddy to optimally sample latent space vectors, with the goal of target molecule generation, we employed the junction tree VAE (JT-VAE) architecture.^91^ The JT-VAE functions as a VAE while encoding and decoding molecular graphs with a high degree of reconstructive accuracy. For this case of targeted molecule generation, we utilized Tversky Similarity^103^ and our own multi-feature objective function to provide fitness metrics for Paddy and Hyperopt (Fig. 5a). For the following trials, we used Pazopanib as the target molecule of interest, and a JT-VAE model trained with the ZINC dataset directly taken from the JT-VAE repo.

We used Tversky (Index) Similarity to compare the associated Morgan Fingerprints^88^ of generated molecules against Pazopanib, and benchmarked both Paddy and Hyperopt against a random sampling algorithm. Tversky Similarity is the generalized form of Tanimoto Similarity, which are both similarity measurers used to compare sets. Tversky Similarity and Tanimoto Similarity differ where *α* and *β* coefficients are set to equal 1 for Tanimoto Similarity and are arbitrary for Tversky Similarity (eqn (12)). For this instance, the sets are the Morgan Fingerprints, with the bit values for hashed subgraphs being the elements. Set *X* was the sampled fingerprint while *Y* was the fingerprint of Pazopanib. The coefficients *α* and *β* were set to 0.5 and 0.01 respectively. The low value *β* was assigned to reduce the penalty for Pazopanib subgraphs not being present in the generated fingerprint.

12 *S*(*X*, *Y*) = |*X*∩*Y*|/(|*X*∩*Y*| + *α*|*X*\*Y*| + *β*|*Y*\*X*|)

The molecule generated by the random sampling algorithm with the greatest fitness was then used as a baseline for comparing the diversity of high similarity molecules generated in turn by Paddy and Hyperopt. This approach for comparing algorithm performance was also employed with the use of our multi-feature objective. To provide further emphasis on drug likeness for generated molecules our custom metric considered in addition to Tversky Similarity: rotatable bonds, the number of cycles, size of cycles, synthetic accessibility, the number of on bits in Morgan Fingerprints, and the number of non-hydrogen atoms (see Methods).

Results from generative sampling of latent space using the two metrics described prior indicated that Paddy is well suited for such a task. Paddy generated molecules with greater maximal fitness, less runtime, and a larger population of molecules outperforming the random search solution than Hyperopt. We found Hyperopt quickly optimizes latent space sampling, though plateauing in performance, whereas Paddy avoids early convergence (Fig. 5b and c). The top scoring molecules generated by both algorithms managed to capture the *m*-toluenesulfonyl moiety of Pazopanib, however the Tversky Similarity metric rewarded generation of molecules with little chemical diversity as to minimize dissimilarity (Fig. 5d), which was mitigated by our custom metric (Fig. 5e). Analysis of the SMILES strings generated by Hyperopt indicates that the algorithm repeatedly samples latent space in the same location after finding a local solution. The convergent behavior of Hyperopt is illustrated by having generated the same solution 249 times using Tversky Similarity (Table 3) and 586 times with our custom metric (Table 4). The SMILES strings of solutions generated by Paddy and Hyperopt can be found in the ESI (Tables S1–6†).

**Table 3:** Performance using Tversky Similarity as objective function

Algorithm	Paddy (population)	Paddy (generational)	Hyperopt	Random
Best solution	0.778	0.776	0.702	0.699
Runtime (seconds)	1365	1441	2232	1171
Total evaluations	4107	3571	3500	3500
Unique solutions	20	25	14	—

**Table 4:** Performance when using custom multifeature objective function

Algorithm	Paddy (population)	Paddy (generational)	Hyperopt	Random
Best solution	2.724	2.265	2.355	1.967
Runtime (seconds)	1317	1849	2120	1170
Total evaluations	3643	5035	3500	3500
Unique solutions	18	33	7	—

Comparing the performance of Paddy, when run in generational mode *versus* population mode, we found the two modes generate differing results while both outperforming Hyperopt. When using Tversky Similarity, the two Paddy modes generated a similar number of unique solutions, though generational mode resulted in solutions of lower similarity with slightly more evaluations and runtime (Table 3). Using our custom metric, generational mode again produced a top solution with a lower score compared to population mode and with a greater runtime. However, generational mode yielded nearly twice the number of unique solutions (Table 4).

**Fig. 6:**
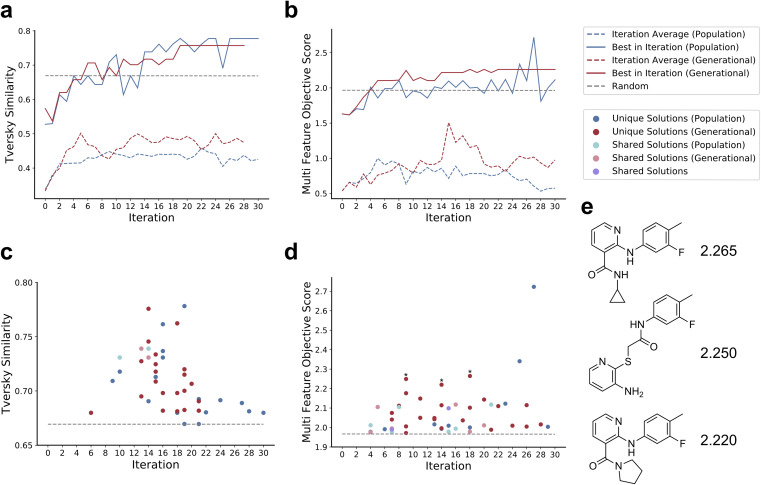
Comparison of Paddy JT-VAE using population versus generational modes. Line plots when using Tversky Similarity (a) and the multi-featured objective function (b) depict the performance as the top score evaluated (solid colored) per iteration, and the average performance per (dashed colored) iteration. Scatter plots for the trials using Tversky Similarity (c) and the multi-featured objective function (d) depict the first instance of generating a molecule of a greater score than the maximal performance from random search. Random search performance values for respective metrics are presented as dashed grey lines. The top three molecules generated in generational mode using the multi-feature objective function (e) are displayed with their respective score and denoted with asterisk on the scatter plot.

For the optimizations using Tversky Similarity the behavior of the two Paddy modes were more so analogous (Fig. 6a). While the average performance per iteration for both modes was nearly identical, the two diverged in terms of top seed performance. Though generational mode produced solutions sooner than population mode, population mode overtook the performance of generational mode halfway through the run. A greater discrepancy in general behavior was observed between the two modes when using the multi-feature custom metric (Fig. 6b). Solutions produced by generational mode displayed a greater average fitness per iteration, and the generational mode run was only to be bested by population mode much closer to the end of the run. As the custom-metric accounts for multiple molecular features, this difference in performance may be a result of population mode being better suited for rapid optimization of relatively smooth response surfaces. Generational mode, however, is inherently more explorative, as it does not sow using the full population of seeds generated during a run. This would lend to the notion of generational mode being better suited for avoiding repeated sampling of local solutions.

**Fig. 7:**
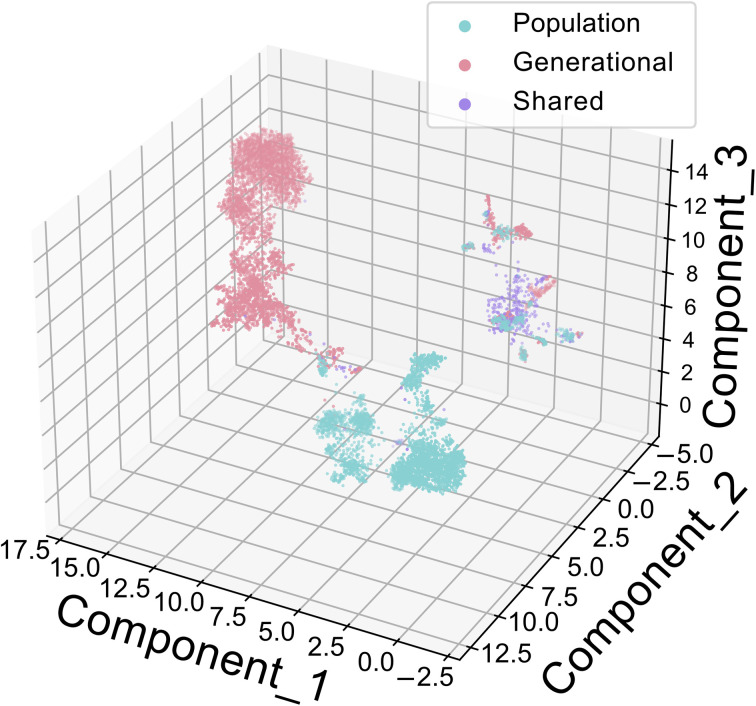
Visualization of the latent space sampled by Paddy in both generational and population mode when using the multi-feature objective function via UMAP. Shared seeds, due to both modes being initiated with the same random variables, are depicted in purple, where generational and population modes are magenta and blue respectively. The greater area sampled by generational mode and its overlap with population mode indicates a greater propensity for explorative behavior during optimization, where population mode appears more exploitive by comparison. This can be visualized best as a rotating gif image provided on GitHub, https://github.com/chopralab/ThePaddyManuscript/tree/master/JTVAE/Plotting/umap.gif.

Comparing the generation of solutions by population and generational mode, previous insight regarding task specific behavior can be further reinforced. Using Tversky Similarity, the two Paddy modes display analogous behavior, as described in prior, with both optimizing similarity between throughout the run (Fig. 6c). Both modes display the same general trends in optimization, generating solutions with increasing fitness while followed by discovery of lower scoring solutions. It is interesting however, to note that there were only two identical solution molecules generated by both algorithms. This low frequency of overlap using Tversky Similarity is contrasted by results from using the multi-feature objective function, where various solutions are both identical and, in some cases, generated by both modes during the same Paddy iteration (Fig. 6d). The overlap in generated solutions would indicate that both Paddy modes sampled latent space in close spatial proximity in part, though with generational mode having sampled both over a larger area and generated a greater number of solutions. A uniform manifold approximation and projection (UMAP)^93^ plot (Fig. 7) supports this, with population mode and generational mode diverging in latent space and generational mode covering a wider area (ESI GIF†).

Overall, these findings support that generational mode is more explorative whereas population mode is more exploitative for sampling. Additionally, multi-feature objective optimization tasks highlight further disparity between number of unique solutions generated by Paddy and Hyperopt optimization methods. We believe this is based on the how these algorithms operate, in that the expected improvement used by Hyperopt is better suited for relatively smooth optimization functions that can be easily modeled *via* Gaussian mixtures. Conversely, the stochastic nature of Paddy is well suited for the rough topology introduced by our multi-feature objective function, as it makes no assumption regarding the underlying function being optimized. This is important for chemical and biological optimization tasks as deviation from an underlying pattern is more useful to explore for discovery.

### Simulated experimental planning

**Fig. 8:**
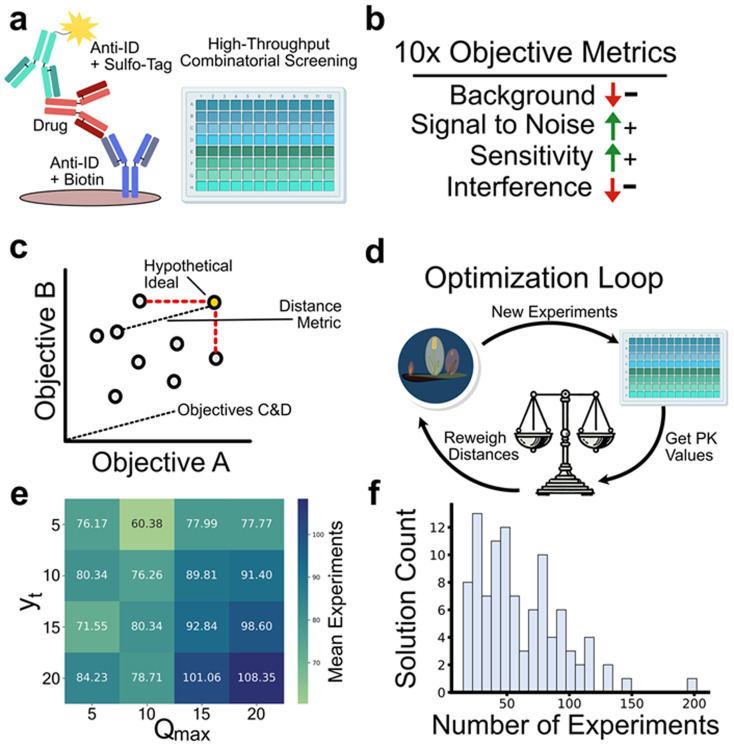
Optimization of pharmacokinetic assay conditions using Paddy. Electrochemiluminescence assays with various combinations of anti-IDs, formats, and concentrations are screened in a high-throughput manner (a). 12 different analytical metrics are used to assess the performance of assay conditions, where the objective is to holistically minimize or maximize certain quantities (b). To quantify performance, the results from screening are used to construct an objective space, where values are normalized and the distance from the ideal combination of values is used to form a distance metric (c). Paddy simulations are conducted where the objective space is regenerated based concurrently with experimental conditions selected by Paddy (d). Simulations with varying *y*_t_ and *Q*_max_ values are used to assess Paddy parameters and their relationship to simulation performance, with the mean number of experiments needed to identify the best set of assay conditions displayed as a heatmap (e). Histogram of 100 repeated simulations, displaying the number of experiments Paddy needs to reach the ideal experimental condition when *y*_t_ and *Q*_max_ are equal to 5 & 10 respectively (f).

While not built directly for self-driving laboratories, the optimization capabilities of Paddy can be extended to real world experimentation. To date, we have utilized Paddy for human-in-the-loop optimization of pulsed valve actuations for introducing reagent into a mass spectrometer for gas-phase ion–molecule reactions (https://github.com/chopralab/cbm_ml_automation).^104^ We have also utilized Paddy for assay optimization based on data collected from real experimentation conducted by members of MSD's Department of Pharmacokinetics, Pharmacodynamics and Drug Metabolism.^17^ Here, we have performed simulated optimization of pharmacokinetic (PK) assays using Paddy to select experimental conditions (Fig. 8) and showcase its performance by modifying user defined parameters, *y*_t_ and *Q*_max_. Briefly, for the PK study, electrochemiluminescence assay conditions were screened such that 360 combinations of, 6 Sulfo-Taged anti-idiotypic antibodies (anti-IDs), 5 biotinylated anti-IDs, 6 assay formats, and 2 soluble antigen concentrations were tested (Fig. 8a). These assay conditions were each evaluated at three different magnitudes of drug concentration, and evaluated for background, upper limit tested sensitivity (100× drug), lower limit tested sensitivity (1× drug), signal to noise (1× drug), and soluble antigen interference (1×, 10×, & 100× drug), totaling to 10 analytical metrics (Fig. 8b). The metric values for a collection of experiments can be thought of in terms of objectives (*i.e.* lower backgrounds and higher sensitivity). By normalizing metric values (with 1 as best and 0 as worst) for a collection of experiments, a hypothetical ideal set of values can be defined as a vector with all elements being 1, with Euclidean distance from the ideal set becoming a singular quantity for minimization (Fig. 8C).

We performed simulations to optimize the conditions for PK assay, where Paddy selects experimental conditions. The distance values are calculated based on the ‘observed’ experiments at time of evaluation (Fig. 8D). Several *y*_t_ and *Q*_max_ parameter values in Paddy were used to further assess the relationship with the mean number of experiments that is required to identify the best set of conditions (Fig. 8E). We found that a general trend of lower *y*_t_ and *Q*_max_ values were associated with less numbers of experiments to reach the best set of assay conditions. Specifically, Paddy reached the best set of conditions with a mean of 60.38 experiments with *y*_t_ = 5 and *Q*_max_ = 10. Interestingly, across 100 repeated trials, we found that fewer experiments can be used to identify optimal results showcasing robustness of Paddy to achieve optimal results, as outlined with benchmarking “toy” examples (Fig. 8F). Overall, these results indicate that Paddy can be used with low values for *y*_t_ and *Q*_max_ to rapidly optimize experimental objectives.

## Summary

We introduce an evolutionary algorithm, Paddy, as a python library containing various methods based on the PFA, for facile, versatile, and robust optimization of numeric parameters for several applications. By considering the spatial distance of parameters and their evaluated performance, Paddy can efficiently optimize a variety of systems without inferring the underlying function. We have benchmarked Paddy against the Tree-structured Parzen Estimator implemented in Hyperopt, Bayesian optimization using a Gaussian process with the Ax platform, and both evolutionary and genetic algorithms with EvoTorch, and we have found Paddy to optimize with low runtime while also avoiding early convergence on a local minimum/maximum.

Meta's Ax and the Chopra Lab's Paddy algorithm both excelled in the benchmark tests, making them strong choices for optimization tasks in chemical sciences. Ax is particularly effective for interpolation problems, while Paddy's low runtime and robustness lends it to being well suited when the search space is entirely unknown. In the context of cheminformatics, we have shown Paddy to perform well with the tasks of hyperparameter optimization and targeted molecule generation. Additionally, we have investigated the differences in behavior between the native PFA Population mode and our variant, Generational mode, and have shown our variant to be better suited for explorative optimization while still retaining general performance. Lastly, we have demonstrated the ability of Paddy to optimize experimental conditions and parameter dependence on search behavior. While Paddy may require more sampling than Bayesian optimization methods, its markedly lower runtime makes it particularly well suited for computational experiments and low cost high-throughput experiments. We believe that these qualities make Paddy well suited for the optimization of chemical systems of high dimensionality and suitable for tasks such as inverse design of drug candidates and autonomous closed-loop experimentation for high to mid throughput experiments. Paddy is open source, and we encourage others to use and improve upon the software to meet their experimental needs.

## Data availability

All data and computer code related to the manuscript is available at https://github.com/chopralab/ThePaddyManuscript (DOI: https://doi.org/10.5281/zenodo.15105999) to encourage others to replicate our work. Complete documentation of the computer code is also available on GitHub at https://github.com/chopralab/paddy (DOI: https://doi.org/10.5281/zenodo.15040660) for others to use and extend Paddy for their chemical optimization tasks.

## Conflicts of interest

Gaurav Chopra is the Director of Merck-Purdue Center funded by Merck Sharp & Dohme LLC., a subsidiary of Merck & Co., Inc., Rahway, NJ, U.S.A. and the co-founder of Meditati Inc., BrainGnosis Inc. and LIPOS BIO Inc. All other authors declare no competing financial interests.

## Supplementary Material

DD-004-D4DD00226A-s001
