# Supplementary material for: Paddy: an evolutionary optimization algorithm for chemical systems and spaces
Source: Digit Discov. 2025 Mar 26;4(5):1352–71. doi: 10.1039/d4dd00226a (PMC12053974; doi:10.1039/d4dd00226a)
Supplement: DD-004-D4DD00226A-s001 [file DD-004-D4DD00226A-s001.pdf]

Supplementary Information (SI) for Digital Discovery.
This journal is © The Royal Society of Chemistry 2025

SUPPORTING INFORMATION

Paddy: Evolutionary Optimization Algorithm for Chemical Systems and Spaces

Armen G. Beck[1†], Sanjay Iyer[1†], Jonathan Fine[1], Gaurav Chopra[1,2,3,4,5,6]

[1]Department of Chemistry and Computer Science (*by courtesy*), Purdue University, 720 Clinic Drive, West Lafayette, IN 47907

[2]Purdue Institute for Drug Discovery, West Lafayette, IN 47907

[3]Purdue Center for Cancer Research, West Lafayette, IN 47907

[4]Purdue Institute for Inflammation, Immunology and Infectious Disease, West Lafayette, IN 47907

[5]Purdue Institute for Integrative Neuroscience, West Lafayette, IN 47907

[6]Regenstrief Center for Healthcare Engineering, West Lafayette, IN 47907

[†]These authors share equal contribution to this work.

*Corresponding author email – gchopra@purdue.edu

Table of Contents

TABLE S1. PADDY (POPULATION) SOLUTIONS USING TVERSKY SIMILARITY AS OBJECTIVE FUNCTION 3

TABLE S2. PADDY (GENERATIONAL) SOLUTIONS USING TVERSKY SIMILARITY AS OBJECTIVE FUNCTION.............. 4

TABLE S3. HYPEROPT SOLUTIONS USING TVERSKY SIMILARITY AS OBJECTIVE FUNCTION 5

TABLE S4. PADDY (POPULATION) SOLUTIONS USING CUSTOM MULTI-FEATURE OBJECTIVE FUNCTION................ 5

TABLE S5. PADDY (GENERATIONAL) SOLUTIONS USING CUSTOM MULTI-FEATURE OBJECTIVE FUNCTION 6

TABLE S6. HYPEROPT SOLUTIONS USING CUSTOM MULTI-FEATURE OBJECTIVE FUNCTION 7

FIGURE S1. GRAMACY & LEE FUNCTION OVERLAID WITH THE BEST RESULT FROM AX OVER 500 TRIALS. 8

FIGURE S2. GRAMACY & LEE FUNCTION OVERLAID WITH THE BEST RESULT FROM PADDY OVER 10 GENERATIONS.. 8

FIGURE S3. GRAMACY & LEE FUNCTION OVERLAID WITH THE BEST RESULT FROM HYPEROPT OVER 1500 TRIALS. 9

FIGURE S4. GRAMACY & LEE FUNCTION OVERLAID WITH THE BEST RESULT FROM EVOTORCH'S EVOLUTIONARY ALGORITHM OVER 10 GENERATIONS. 9

FIGURE S5. GRAMACY & LEE FUNCTION OVERLAID WITH THE BEST RESULT FROM EVOTORCH'S GENETIC ALGORITHM OVER 10 GENERATIONS. 10

FIGURE S6. GRAMACY & LEE FUNCTION OVERLAID WITH THE BEST RESULT FROM RANDOM PREDICTIONS OVER 1500 TRIALS. 10

FIGURE S7. HEATMAP OF BEST MEAN SQUARED ERROR (MSE) ACROSS 5 REPEATED RUNS FROM GRAMACY & LEE OPTIMIZATION GRID SEARCH.. 11

FIGURE S8. HEATMAP OF BEST F1 SCORES ACROSS 5 REPEATED RUNS FROM MLP HYPERPARAMETER OPTIMIZATION GRID SEARCH. 12

Table S1. Paddy (Population) Solutions using Tversky Similarity as Objective Function

SMILE string	score	frequency
Cc1ccc(NC(=O)c2ccncc2)cc1S(N)(=O)=O	0.778210116732	8
Cc1ccc(NC(=O)C2CC2)cc1S(N)(=O)=O	0.762300762301	1
Cc1ccc(NC(=O)c2ccccn2)cc1S(N)(=O)=O	0.761498629302	15
[α] Cc1ccc(NC(=O)c2cccnc2N)cc1S(N)(=O)=O	0.738989062962	12
Cc1ccc(NC(=O)c2ccc(F)cc2)cc1S(N)(=O)=O	0.736771600804	2
Cc1ccc(NC(=O)c2cccnc2)cc1S(N)(=O)=O	0.730816077954	16
[α] Cc1ccc(NC(=O)c2ccc(N)cn2)cc1S(N)(=O)=O	0.730816077954	1
Cc1ccc(NC(=O)c2ccc3ccccc3n2)cc1S(N)(=O)=O	0.717772035601	3
Cc1ccc(NC(=O)c2ccccc2F)cc1S(N)(=O)=O	0.712896953986	3
Cc1ccc(NC(=O)c2ccnc(N(C)C)c2)cc1S(C)(=O)=O	0.709219858156	1
Cc1cc(NC(=O)c2ccncc2)ccc1S(N)(=O)=O	0.692520775623	3
Cc1ccc(NC(=O)c2ccncc2)cc1S(=O)(=O)N(C)C	0.691471847218	7
Cc1ccc(NC(=O)c2ccnc(N)c2)cc1S(C)(=O)=O	0.690521029504	1
Cc1ccc(NC(=O)c2ccc(C#N)cn2)cc1S(N)(=O)=O	0.688863375431	1
Cc1ccc(NC(=O)c2ccncc2)cc1S(C)(=O)=O	0.681247759053	2
Cc1cc(NC(=O)c2ccccn2)ccc1S(N)(=O)=O	0.680272108844	1
Cc1ccc(NC(=O)c2ccc[nH]c2=O)cc1S(N)(=O)=O	0.679851668727	5
Cc1ccc(NC(=O)c2ccccn2)cc1S(=O)(=O)N(C)C	0.679851668727	1
Cc1ccc(NC(=O)c2cccnc2N)cc1S(=O)(=O)N(C)C	0.669577874818	1
Cc1ccc(NC(=O)c2ccc(N(C)C)nc2)cc1S(C)(=O)=O	0.669506999391	1

[α]SMILES generated using both Paddy types.

Table S2. Paddy (Generational) Solutions using Tversky Similarity as Objective Function

SMILES string	score	frequency
Cc1cnc(Nc2ccc(S(N)(=O)=O)cc2)nc1C	0.775740479549	47
Cc1cnc(Nc2ccc(S(N)(=O)=O)cc2)nc1N	0.762300762301	1
Cc1cnc(Nc2cccc(S(N)(=O)=O)c2)nc1C	0.745542949757	6
[α] Cc1ccc(NC(=O)c2cccnc2N)cc1S(N)(=O)=O	0.738989062962	2
Cc1cnc(Nc2cccc(S(N)(=O)=O)c2)nc1N	0.7336523126	1
[α] Cc1ccc(NC(=O)c2ccc(N)cn2)cc1S(N)(=O)=O	0.730816077954	1
Cc1cnc(Nc2ccccc2S(N)(=O)=O)nc1C	0.727398683755	54
Cc1ccc(NC(=O)c2ccccc2N)cc1S(N)(=O)=O	0.724637681159	1
Cc1cnc(Nc2ccc(S(C)(=O)=O)cc2)nc1C	0.719969685487	1
Cc1cnc(Nc2ccc(NS(C)(=O)=O)cc2)nc1C	0.719969685487	1
CCn1nccc1C(=O)Nc1ccc(C)c(S(N)(=O)=O)c1	0.717772035601	1
Cc1cnc(Nc2ccccc2S(N)(=O)=O)nc1N	0.715015321757	1
Cc1cnc(Nc2ccc(S(=O)(=O)N(C)C)cc2)nc1	0.708661417323	1
Cc1ncc(C)c(Nc2ccc(S(N)(=O)=O)cc2)n1	0.706582372629	1
Cc1cc(NC(=O)c2nccn2C)ccc1S(N)(=O)=O	0.700152207002	1
Cc1cc(Nc2ccc(S(N)(=O)=O)cc2)nc(C)n1	0.694980694981	46
Cc1ccccc1S(=O)(=O)Nc1ccccc1S(N)(=O)=O	0.698080279232	3
Cc1cnc(Nc2ccc(NS(C)(=O)=O)cc2)nc1	0.698080279232	2
Cc1ccc(NC(=O)c2nccn2C)cc1S(C)(=O)=O	0.690521029504	1
Cc1ncnc(C)c1Nc1ccccc1S(N)(=O)=O	0.682456844641	1
Cc1nc(N)cc(Nc2ccc(S(N)(=O)=O)cc2)n1	0.681818181818	4
Cc1ncc(Nc2ccc(S(N)(=O)=O)cc2)c(C)n1	0.681818181818	1
Cc1ccccc1S(=O)(=O)Nc1ncc(N(C)C)cn1	0.681818181818	1
Cc1ncc(C)c(Nc2ccccc2S(N)(=O)=O)n1	0.681247759053	1
Cc1ccc(NC(=O)c2ccc(=O)n(C)n2)cc1S(C)(=O)=O	0.679851668727	5

[α]SMILES generated using both Paddy types.

Table S3. Hyperopt Solutions using Tversky Similarity as Objective Function

SMILES string	score	frequency
Cc1ccc(NC(=O)CC2CCCC2)cc1S(N)(=O)=O	0.701530612245	249
Cc1ccc(NC(=O)Cc2ccc(F)cc2)cc1S(N)(=O)=O	0.701530612245	3
Cc1ccc(NC(=O)Cc2ccc(Cl)cc2)cc1S(N)(=O)=O	0.701530612245	2
Cc1ccc(NC(=O)Cc2cccs2)cc1S(N)(=O)=O	0.679851668727	29
Cc1ccc(NC(=O)CCC2CCCC2)cc1S(N)(=O)=O	0.679851668727	20
Cc1ccc(NC(=O)Cc2ccsc2)cc1S(N)(=O)=O	0.679851668727	10
Cc1ccc(NC(=O)Cc2ccccc2F)cc1S(N)(=O)=O	0.679851668727	7
Cc1ccc(NC(=O)Cc2ccc[nH]2)cc1S(N)(=O)=O	0.679851668727	2
Cc1ccc(NC(=O)Cc2ccccc2Cl)cc1S(N)(=O)=O	0.679851668727	2
Cc1ccc(NC(=O)C2CC=CCC2)cc1S(N)(=O)=O	0.679851668727	1
Cc1ccc(NC(=O)CCSc2ccccn2)cc1S(N)(=O)=O	0.678794461037	1
Cc1ccc(NC(=O)Cc2cccc(F)c2)cc1S(N)(=O)=O	0.669506999391	18
Cc1ccc(NC(=O)Cc2cccc(Cl)c2)cc1S(N)(=O)=O	0.669506999391	3
Cc1ccc(NCc2cc(C#N)cs2)cc1S(N)(=O)=O	0.669506999391	2

Table S4. Paddy (Population) Solutions using Custom Multi-Feature Objective Function

SMILES string	score	frequency
Cc1ccc(NC(=O)c2cccc3ncccc23)cc1S(N)(=O)=O	2.723916711	1
Cc1ccc(NC(=O)c2cccc3ncccc23)cc1S(C)(=O)=O	2.34155450409	1
Cc1ccc(NC(=O)c2cc(C3CC3)[nH]n2)cc1S(N)(=O)=O	2.12329223522	1
$^{\alpha}$ Cc1ccc(NC(=O)c2cccnc2N2CCCC2)cc1Cl	2.11849336272	2
$^{\alpha}$ Cc1ccc(NC(=O)c2cccnc2N2CCCC2)cc1F	2.10555596297	5
$^{\alpha}$ Cc1ccc(Nc2ncccc2C(=O)N2CCOCC2)cc1F	2.0994818624	1
Cc1ccc(NC(=O)c2ccccn2)cc1N1CCCC1=O	2.01674640789	2
$^{\alpha}$ Cc1ccc(NC(=O)C2CC2)cc1Nc1ncccc1C#N	2.01188802674	1
Cc1ccc(NC(=O)c2cccc3ncccc23)cc1-n1cnnn1	2.00986738561	4
Cc1ccc(S(C)(=O)=O)cc1NC(=O)c1cccc2ncccc12	2.0047763139	1
Cc1ccc(NC(=O)c2ccnc(-n3ccnc3)c2)cc1Cl	2.00087779538	1
$^{\alpha}$ Cc1ccc(NC(=O)c2cccnc2N2CCOCC2)cc1F	1.99455379655	2
Cc1ccc(NC(=O)c2ccccn2)cc1-n1cnnn1	1.99378763988	3
Cc1ccc(Cl)cc1NC(=O)c1cccnc1N1CCCC1	1.99152386104	1
$^{\alpha}$ Cc1ccc(NC(=O)c2ccnc(-n3ccnc3)c2)cc1F	1.99001940011	5
$^{\alpha}$ Cc1cc(NC(=O)c2cccnc2N2CCCC2)ccc1F	1.97918828426	1
Cc1cccc(NC(=O)Cn2cnc3c(cnn3C)c2=O)c1	1.97823445804	7
$^{\alpha}$ Cc1ccc(F)cc1NC(=O)c1cccnc1N1CCCC1	1.9762861328	1

$^{\alpha}$SMILES generated using both Paddy types.

Table S5. Paddy (Generational) Solutions using Custom Multi-Feature Objective Function

SMILES string	score	frequency
Cc1ccc(Nc2ncccc2C(=O)NC2CC2)cc1F	2.2654205205	40
Cc1ccc(NC(=O)CSc2ncccc2N)cc1F	2.24971825997	1
Cc1ccc(Nc2ncccc2C(=O)N2CCCC2)cc1F	2.22047936415	103
Cc1ccc(NC(=O)C2CC2)cc1NCc1ccccn1	2.17662029154	1
[β] Cc1ccc(NC(=O)NCc2cccnc2)cc1S(C)(=O)=O	2.14919940829	1
Cc1ccc(Nc2nc(C(=O)N3CCCC3)cs2)cc1F	2.14446609203	2
[α] Cc1ccc(NC(=O)c2cccnc2N2CCCC2)cc1Cl	2.11849336272	4
Cc1ccc(Nc2ncccc2C(=O)N2CCCCC2)cc1F	2.1159438347	3
Cc1cc(NC(=O)COc2cccc(F)c2)cc2ncccc12	2.11492395855	1
Cc1cc(NC(=O)Cn2ccccc2=O)cc2ncccc12	2.11229538267	1
Cc1ccc(Nc2ncccc2C(=O)N2CCOCC2)cc1Cl	2.11082181245	1
[α] Cc1ccc(NC(=O)c2cccnc2N2CCCC2)cc1F	2.10555596297	313
Cc1cc(Nc2ncccc2C(=O)N2CCCC2)ccc1F	2.10255102342	3
[α] Cc1ccc(Nc2ncccc2C(=O)N2CCOCC2)cc1F	2.0994818624	11
Cc1cccc(Nc2cc(C(=O)N3CCOCC3)ccn2)c1	2.07514478583	1
Cc1ccc(NC(=O)NCCc2cccnc2)cc1S(C)(=O)=O	2.05021322347	1
Cc1ccc(NC(=O)c2ccnc(N3CCOCC3)c2)cc1F	2.0491883323	5
Cc1ccc(Nc2ncccc2C(=O)N2CCOCC2)cc1C	2.04323215196	4
Cc1ccc(NCc2cccnc2)cc1N1CCCC1=O	2.04115602872	1
Cc1ccc(Nc2ncccc2C(=O)NC2CCCC2)cc1F	2.03727815308	2
Cc1ccc(NC(=O)c2ccnc(-n3cncn3)c2)cc1Cl	2.01691530646	1
[α] Cc1ccc(NC(=O)C2CC2)cc1Nc1ncccc1C#N	2.01188802674	2
Cc1cccnc1CNC(=O)Nc1ccc2ncsc2c1	2.01038606454	1
Cc1ccc(NC(=O)c2ccnc(-n3cncn3)c2)cc1F	2.0060616961	5
Cc1ccc(NC(=O)c2cccnc2N2CCOCC2)cc1Cl	2.0057323405	1
Cc1ccc(NC(=O)c2cc(N3CCOCC3)ccn2)cc1F	1.99676506225	1
Cc1cccc(Nc2ncccc2C(=O)N2CCOCC2)c1	1.99517654393	3
[α] Cc1ccc(NC(=O)c2cccnc2N2CCOCC2)cc1F	1.99455379655	43
[α] Cc1ccc(NC(=O)c2ccnc(-n3ccnc3)c2)cc1F	1.99001940011	13
Cc1ccc(N)cc1NC(=O)c1cccnc1N1CCCC1	1.98938417372	1
[α] Cc1cc(NC(=O)c2cccnc2N2CCCC2)ccc1F	1.97918828426	2
[α] Cc1ccc(F)cc1NC(=O)c1cccnc1N1CCCC1	1.9762861328	11
Cc1ccc(NC(=O)c2cccnc2)cc1N1CCCC1=O	1.97188960395	1

[α]SMILES generated using both Paddy types, [β]SMILES generated using both Paddy (generational) and Hyperopt.

Table S6. Hyperopt Solutions using Custom Multi-Feature Objective Function

SMILES string	score	frequency
Cc1ccc(NC(=O)CSc2nccc(N)n2)cc1Cl	2.35489428064	586
Cc1ccc(Cl)cc1NC(=O)CSc1nccc(N)n1	2.25171293618	5
Cc1ccc(NC(=O)CCn2cccn2)cc1S(C)(=O)=O	2.19219435007	2
Cc1ccc(OCC(=O)Nc2cccc3ncccc23)cc1C	2.17151074779	3
[β] Cc1ccc(NC(=O)NCc2cccnc2)cc1S(C)(=O)=O	2.14919940829	1
Cc1ccc(NC(=O)CC2CCCO2)cc1S(N)(=O)=O	2.09813037529	1
CC(=O)Nc1cccc(CNC(=O)c2ccc3c(C)ccnc3c2)c1	2.00645535494	1

[β]SMILES generated using both Paddy (generational) and Hyperopt.

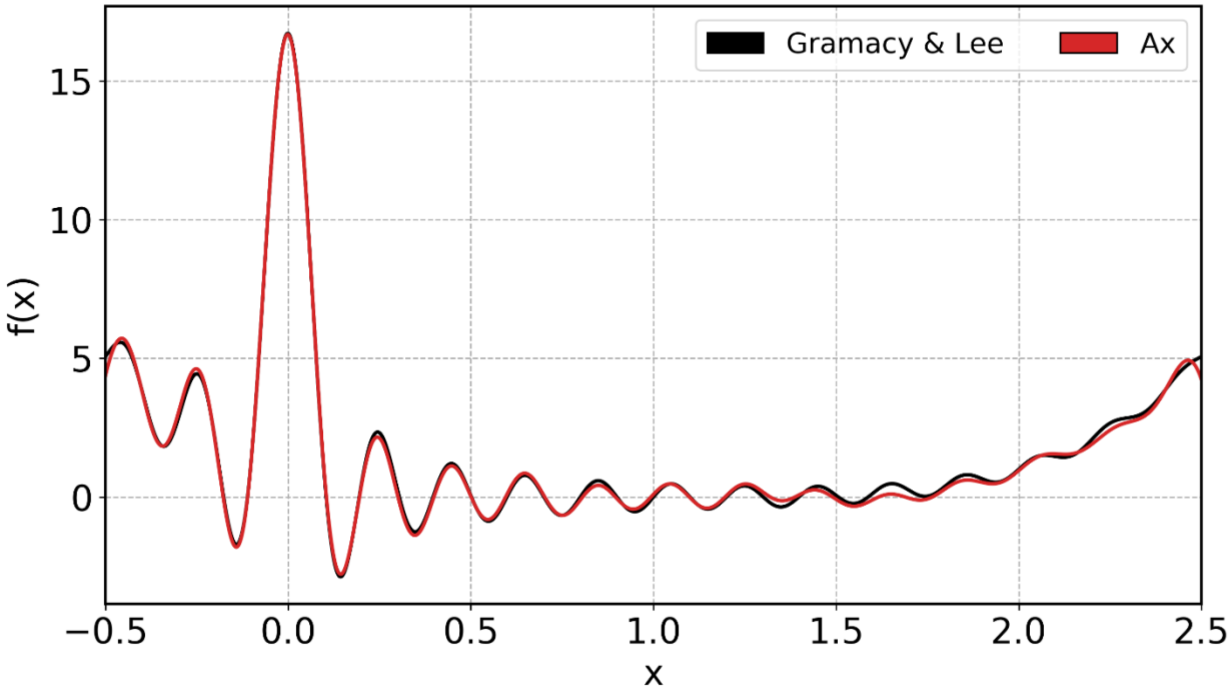

Figure S1. Gramacy & Lee Function overlaid with the best result from Ax over 500 trials.

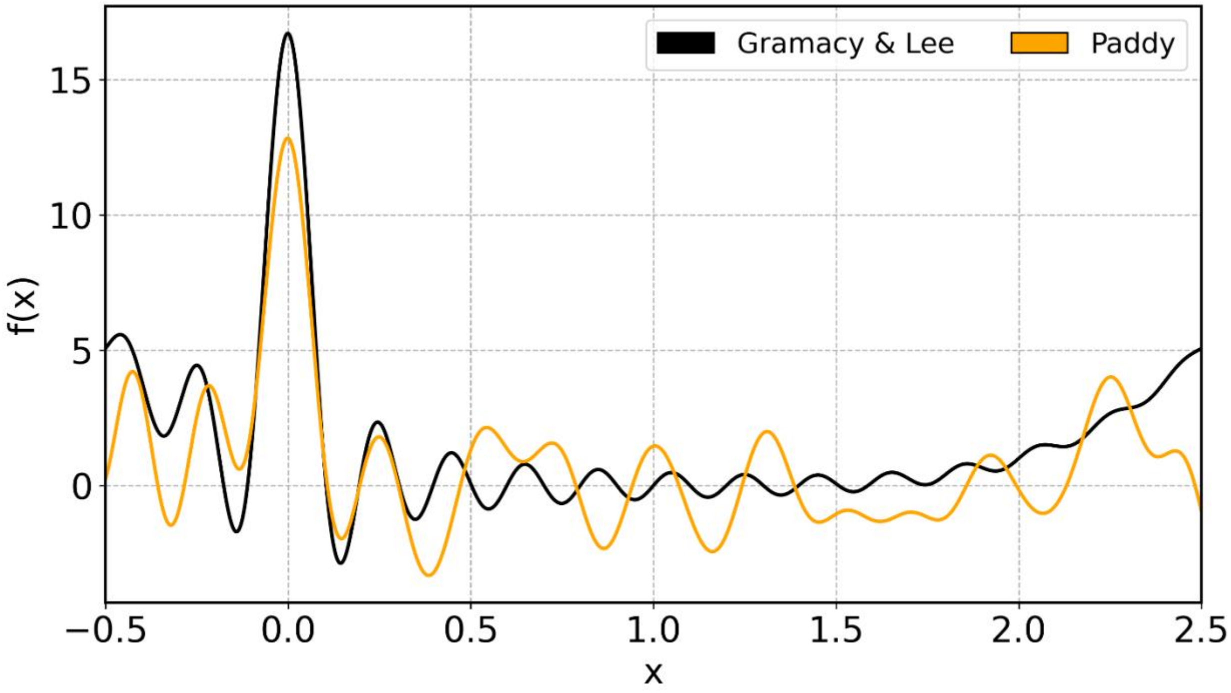

Figure S2. Gramacy & Lee Function overlaid with the best result from Paddy over 10 generations.

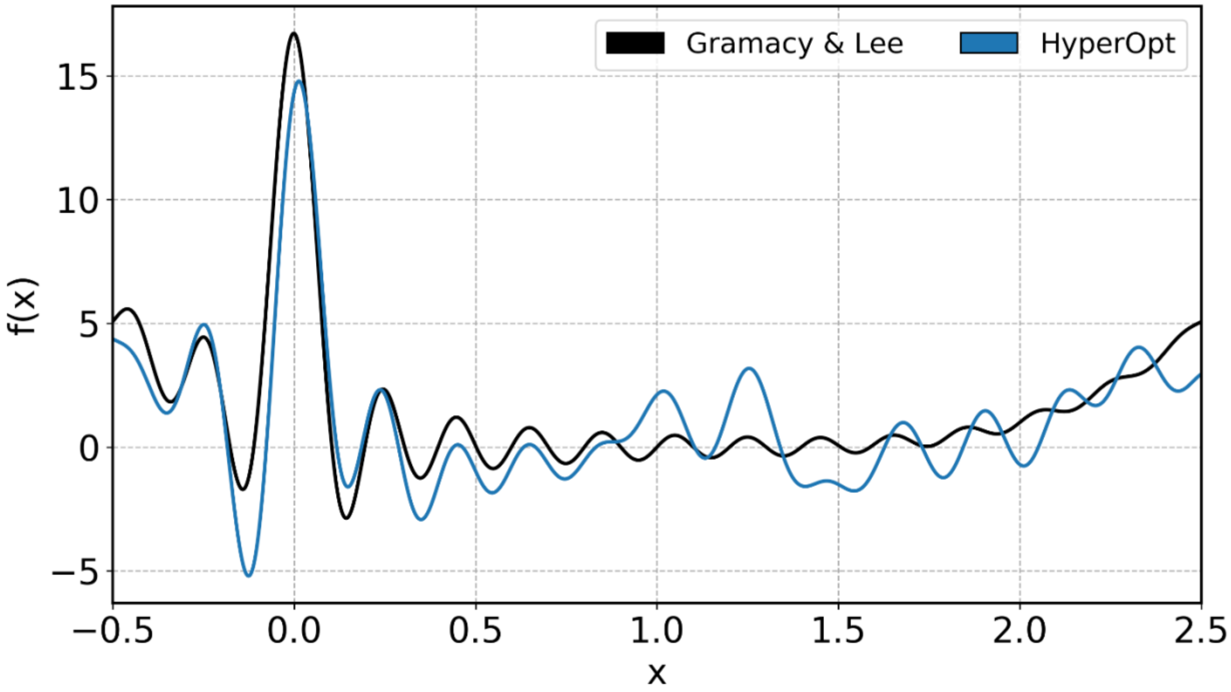

Figure S3. Gramacy & Lee Function overlaid with the best result from HyperOpt over 1500 trials.

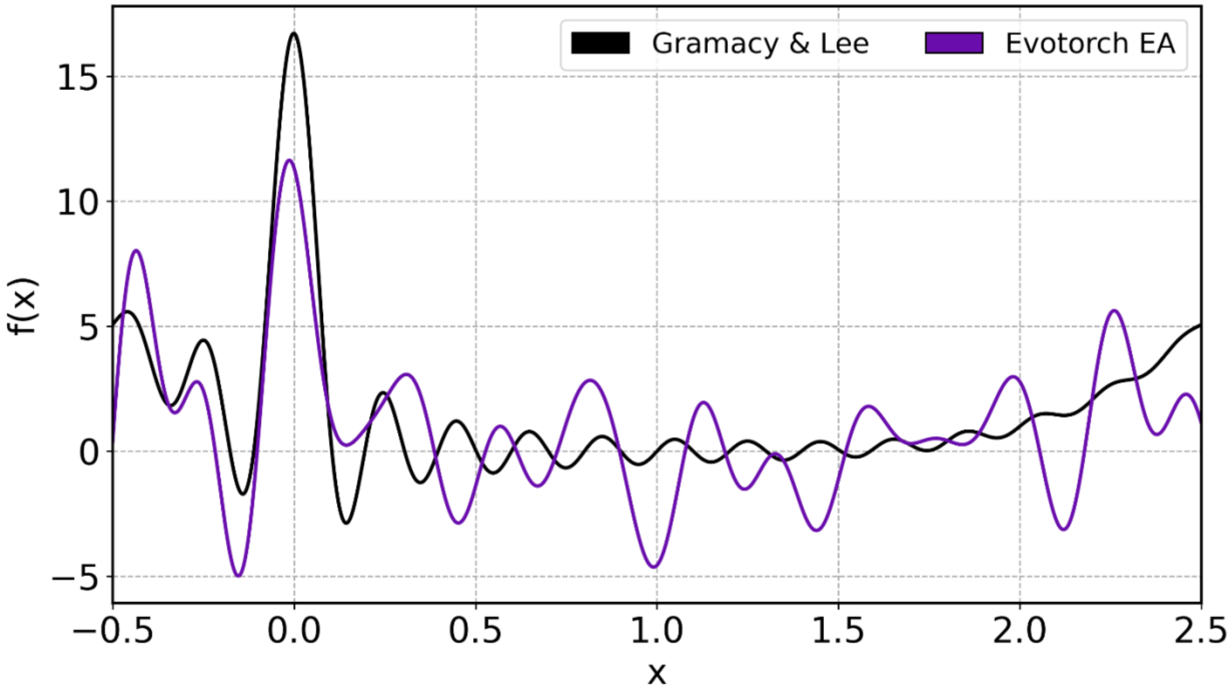

Figure S4. Gramacy & Lee Function overlaid with the best result from EvoTorch's Evolutionary Algorithm over 10 generations.

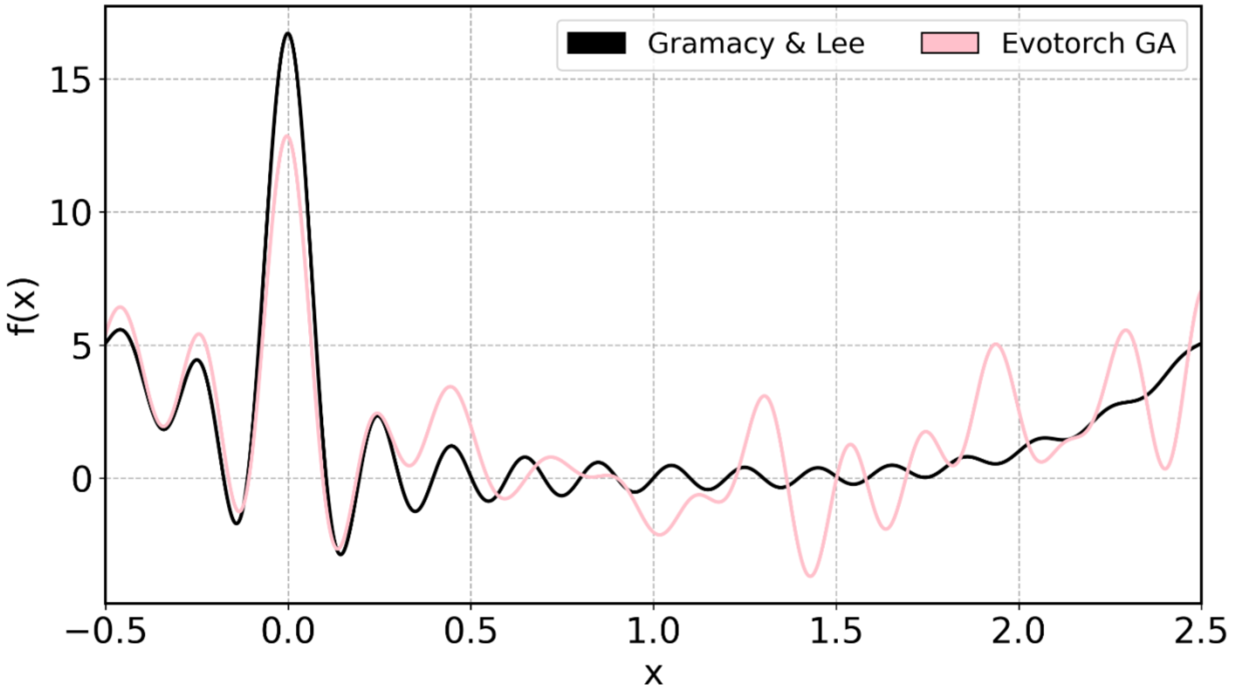

Figure S5. Gramacy & Lee Function overlaid with the best result from EvoTorch's Genetic Algorithm over 10 generations.

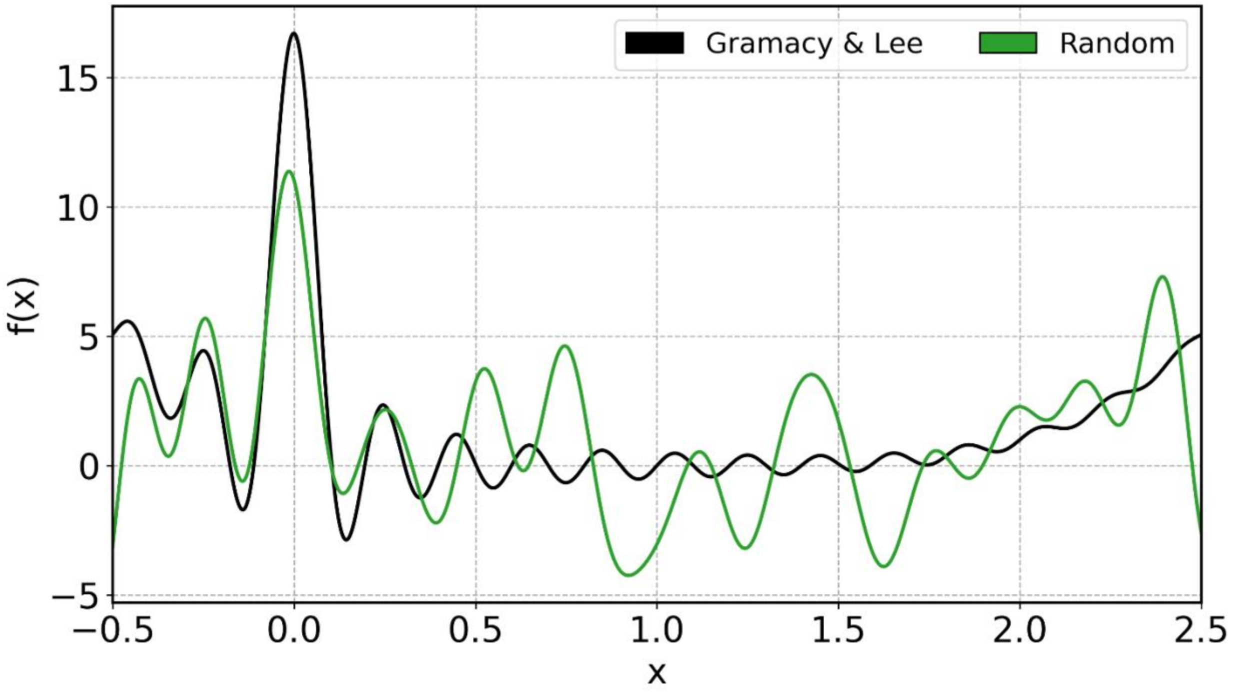

Figure S6. Gramacy & Lee Function overlaid with the best result from random predictions over 1500 trials.

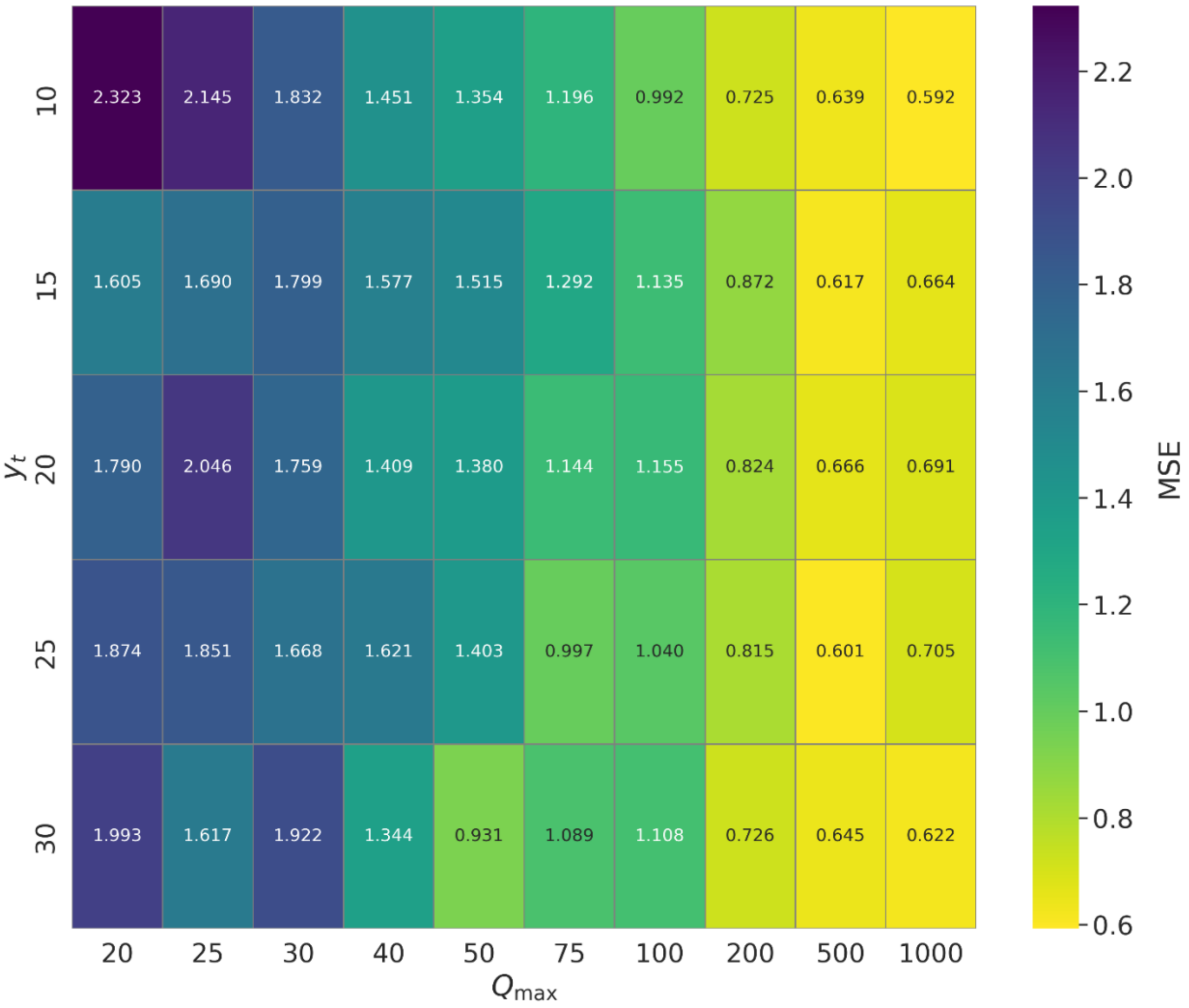

Figure S7. Heatmap of best Mean Squared Error (MSE) across 5 repeated runs from Gramacy & Lee optimization grid search. Y-axis shows y_t (yield threshold), which determines the minimum fitness threshold for plant selection. X-axis shows Q_{max} (maximum seed quota), which sets the upper limit of seeds each selected plant can produce. Experiments utilized Paddy's generational mode with default Gaussian dispersion settings.

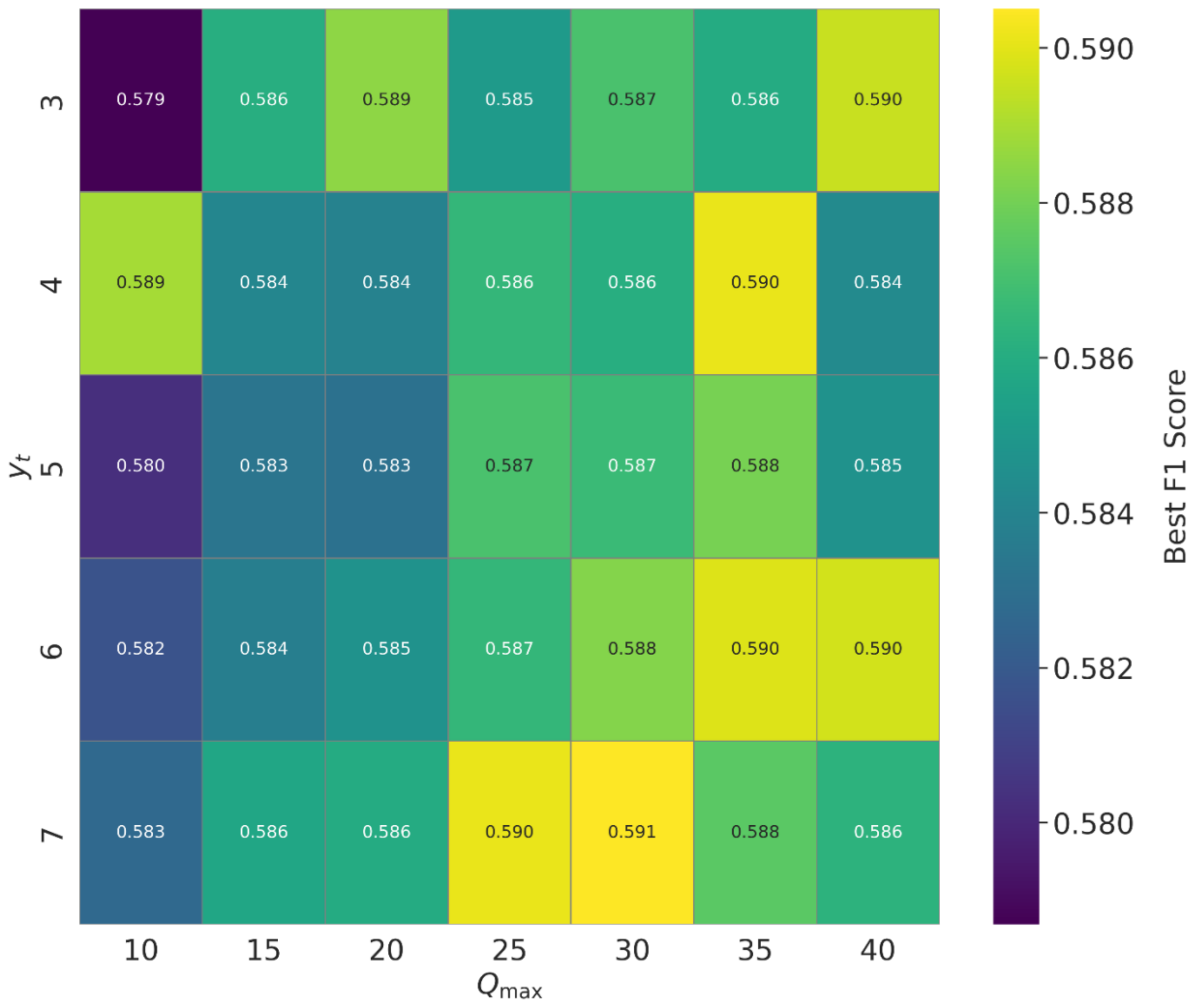

Figure S8. Heatmap of best F1 scores across 5 repeated runs from MLP hyperparameter optimization grid search. Y-axis shows y_t (yield threshold), which determines the minimum fitness threshold for plant selection. X-axis shows Q_{max} (maximum seed quota), which sets the upper limit of seeds each selected plant can produce. Experiments utilized Paddy's generational mode with default Gaussian dispersion settings.
